# Metabolic Profiling Reveals Potential Prognostic Biomarkers for SFTS: Insights into Disease Severity and Clinical Outcomes

**DOI:** 10.3390/metabo15040228

**Published:** 2025-03-27

**Authors:** Zhuo-Min Zhu, Huan-Yu Liu, Na An, An-Ling Li, Jia Li, Sai-Jun Wang, Gui Yang, Yong-Wei Duan, Ying Yang, Mei Zhang, Quan-Fei Zhu, Song-Mei Liu, Yu-Qi Feng

**Affiliations:** 1 School of Bioengineering and Health, Wuhan Textile University, Wuhan 430200, China; 2023283050058@whu.edu.cn (Z.-M.Z.); 2017202030018@whu.edu.cn (N.A.); jiajiali@whu.edu.cn (J.L.); yqfeng@whu.edu.cn (Y.-Q.F.); 2 School of Public Health, Wuhan University, Wuhan 430072, China; 2023283050079@whu.edu.cn; 3 Department of Clinical Laboratory, Center for Gene Diagnosis & Program of Clinical Laboratory, Zhongnan Hospital of Wuhan University, Wuhan 430071, China; lhuanyu@whu.edu.cn (H.-Y.L.); zn000843@whu.edu.cn (A.-L.L.); yangg_whu@whu.edu.cn (G.Y.); 2010302210025@whu.edu.cn (Y.-W.D.); yangying0109@whu.edu.cn (Y.Y.); 4 Department of Obstetrics, Zhongnan Hospital of Wuhan University, Wuhan 430071, China; 5 Department of Clinical Laboratory, Ezhou Hospital of Traditional Chinese Medicine, Ezhou 436000, China; gzhzm817@sina.com; 6 Hubei Province Key Laboratory of Allergy and Immunology, School of Basic Medical Sciences, Wuhan University, Wuhan 430071, China; 7 Frontier Science Center for Immunology and Metabolism, Wuhan University, Wuhan 430071, China

**Keywords:** severe fever with thrombocytopenia syndrome, prognostic biomarker, metabolomics, LC-MS

## Abstract

**Background/Objectives:** Severe fever with thrombocytopenia syndrome (SFTS) is a viral infection primarily found in Asia, with a case fatality rate of about 10%. Despite its increasing prevalence, the underlying pathogenic mechanisms remain poorly understood, limiting the development of effective therapeutic interventions. **Methods:** We employed an untargeted metabolomics approach using liquid chromatography–mass spectrometry (LC-MS) to analyze serum samples from 78 SFTS patients during the acute phase of their illness. Differential metabolic features between survival and fatal cases were identified through multivariate statistical analysis. Furthermore, we constructed a metabolic prognostic model based on these biomarkers to predict disease severity. **Results:** Significant alterations were observed in four key metabolic pathways: sphingolipid metabolism, biosynthesis of phenylalanine, tyrosine, and tryptophan, primary bile acid biosynthesis, and phenylalanine metabolism. Elevated levels of phenyllactic acid and isocitric acid were strongly associated with adverse outcomes and demonstrated high discriminatory power in distinguishing fatal cases from survivors. The metabolic prognostic model incorporating these biomarkers achieved a sensitivity of 75% and a specificity of 90% in predicting disease severity. **Conclusions:** Our findings highlight the pivotal role of metabolic dysregulation in the pathogenesis of SFTS and suggest that targeting specific metabolic pathways could open new avenues for therapeutic development. The identification of prognostic biomarkers provides a valuable tool for early risk stratification and timely clinical intervention, potentially improving patient outcomes.

## 1. Introduction

Severe fever with thrombocytopenia syndrome (SFTS) is an emerging hemorrhagic fever caused by infection with the novel Bunyavirus (SFTSV). This zoonotic disease, primarily transmitted through tick bites, is characterized by high fever, leukopenia, and thrombocytopenia. Severe cases can lead to multi-organ failure, hemorrhage, and death [1,2,3]. Since its initial identification in 2009, the number of reported cases has been increasing annually, with a notable prevalence in Asian regions, including China, Japan, and Korea [4,5,6,7]. The disease predominantly occurs from April to October, with a peak incidence from May to July [8]. In endemic areas, such as northeastern China and the rural mountainous and hilly regions of central and eastern China [9], the infection rate of this virus ranges from 1% to 3%, with a fatality rate varying between 6% and 30% across different studies, averaging around 10% [1,3,7,10,11]. Currently, SFTS has emerged as a significant public health concern. In 2017, the World Health Organization (WHO) listed SFTS among the global priority infectious diseases.

Early diagnosis of SFTS is crucial for improving patient outcomes and preventing disease transmission. Despite the availability of various PCR techniques for rapid diagnosis [12,13], there are currently no specific drugs or effective vaccines targeting SFTSV. The primary treatment approach for SFTSV-infected patients remains symptomatic supportive care and broad-spectrum antiviral therapy [14,15]. Therefore, the treatment of SFTS patients remains challenging. Timely intervention before the disease progresses to its acute phase can effectively control its progression. Therefore, early diagnosis of severe patients is essential, enabling clinical teams to respond quickly, intervene, seize the optimal treatment window, enhance patient outcomes, and reduce mortality. Additionally, the incomplete understanding of SFTS pathogenesis significantly hinders the development of effective treatments and preventive measures.

Viral infections possess the ability to reprogram host metabolism, creating an environment that facilitates their replication and survival [16,17]. Changes in serum metabolites can serve as indicators of systemic metabolic alterations driven by persistent viral replication. Metabolomics is a field of study that encompasses a range of analytical techniques to capture real-time, comprehensive changes in small molecules within biofluids, cells, and tissues [18]. In recent years, the metabolomics analysis of host serum has emerged as a promising tool for elucidating host–virus interactions in vivo and identifying diagnostic biomarkers. This methodology has already yielded significant insights into the pathogenesis of infections, such as SARS-CoV-2 and dengue virus (DENV) [19,20,21]. Several previous studies have investigated metabolite changes in hosts following SFTSV infection using metabolomics techniques. For example, Li et al. identified that disruptions in the arginine catabolism pathway were linked to platelet homeostasis and T-cell dysregulation following SFTSV infection [22]. Zhang and colleagues observed disturbances in tryptophan and phenylalanine metabolism in the urine of SFTSV-infected patients [23]. However, research on metabolic alterations in SFTS patients is still limited. Moreover, metabolic disorders during disease progression form a dynamically evolving network, and studies examining the metabolic status throughout the course of SFTS are particularly scarce. Consequently, further investigation into the metabolic changes in SFTS patients, including the precise characterization of the subtle differences and dynamic trajectories, is essential for elucidating disease mechanisms and developing potential therapeutic strategies.

**Figure 1:**
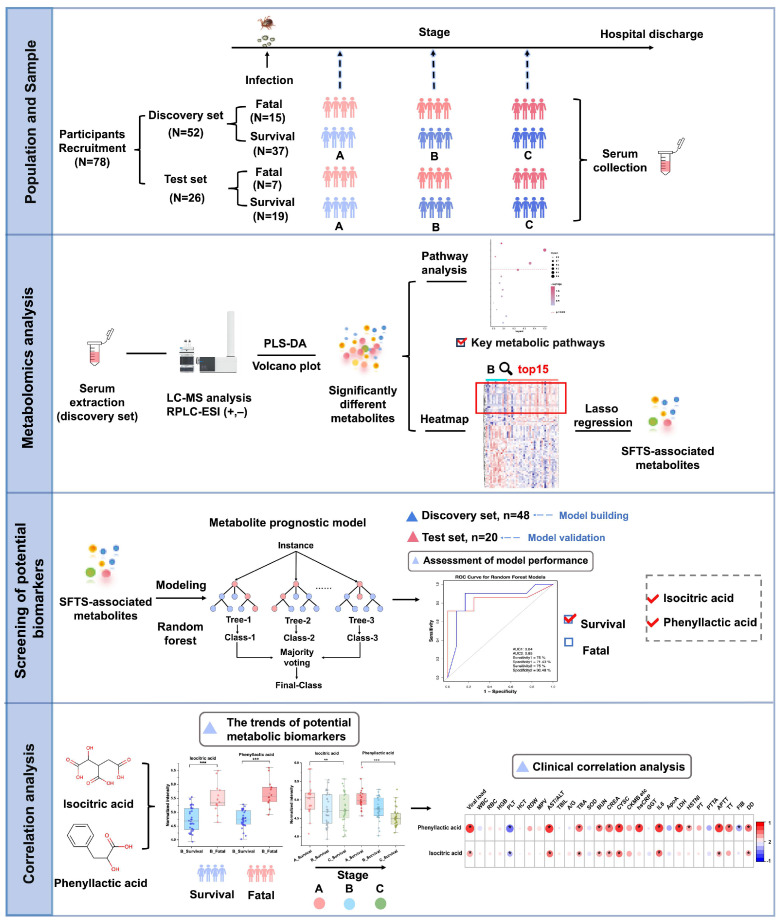
Schematic overview of the study design. The study encompassed a total of 78 participants, divided into a discovery set of 52 individuals and a test set of 26 individuals. Patients were classified into three clinical stages (A, B, and C) according to disease progression, and non-targeted metabolomics analysis was performed on their serum samples. Metabolic profiles were compared between patients with survival and fatal outcomes to elucidate metabolic reprogramming in severe disease states and identify altered metabolic pathways. A metabolic prognostic model, incorporating phenyllactic acid and isocitric acid, was developed using machine learning techniques based on the discovery cohort data and subsequently validated in the test set. Longitudinal and cross-sectional trends of the prognostic metabolites were analyzed and correlated with clinical data.

In this study, we utilized a liquid chromatography–mass spectrometry (LC-MS)-based untargeted metabolomics approach to analyze serum samples from 78 SFTS patients (discovery set: 52 patients; test set: 26 patients) at multiple time points from admission to discharge, as depicted in Figure 1. Our primary objective was to comprehensively characterize the metabolic alterations in SFTSV-infected patients throughout the entire disease course. To achieve this, we applied two grouping strategies. First, we categorized the patients into survival and fatal groups based on their final outcomes. By comparing the serum metabolite profiles between these groups, we aimed to unveil the impact of SFTSV on host metabolism, identify abnormal metabolic pathways and significantly altered metabolites, and explore the potential pathogenic mechanisms of the virus. These findings were used to develop a metabolic prognostic model to aid clinicians in the early diagnosis of severe cases and improve patient outcomes. Second, we divided the population into three stages (stages A, B, and C) based on three critical time points in disease progression, focusing on the temporal changes in metabolites. From a metabolomics standpoint, we delineated the metabolic features of SFTSV-infected patients as their condition evolved, examined the pathogenic characteristics of the virus, and offered novel insights into the infection process and pathogenic mechanisms of SFTSV.

## 2. Materials and Methods

### 2.1. Study Population Information

A total of 78 patients diagnosed with SFTS were enrolled at the Zhongnan Hospital of Wuhan University between June 2021 and October 2023. This cohort included 52 patients in the discovery set (15 fatal and 37 survival cases) and 26 patients in the test set (7 fatal and 19 survival cases). Inclusion criteria were as follows: in-hospital patients coded with disease A93.802, acute fever, thrombocytopenia, and positive RT-qPCR for SFTSV RNA. Exclusion criteria included outpatients, patients admitted for ≤3 days, and those lost to follow-up. Table S1 presents information on other common viral infections in the discovery set, including Hepatitis B virus (HBV), Epstein–Barr virus (EBV), Influenza A virus (FluA), and Influenza B virus (FluB). In the test set, one case in the survival group and one case in the fatal group were associated with EBV infection. The positive rates of these viruses did not differ between the survival and fatal groups, either in the discovery set or in the test set (all *p* > 0.05). All patients met the diagnostic criteria for SFTS and were confirmed by RT-qPCR detection of SFTSV RNA. Serum viral loads of SFTSV were determined by commercial kits using RT-qPCR (Daan, Guangzhou, China), following the manufacturer’s instructions. Clinical data were retrospectively collected from medical records, following a standardized protocol. Clinical outcomes were followed up until 28 February 2024. The study received approval from the Medical Ethics Committee of Zhongnan Hospital of Wuhan University (approval number: 2024046K). All procedures involving human participants adhered to the principles outlined in the Declaration of Helsinki.

### 2.2. Chemicals and Reagents

Metabolite standards were purchased from Aladdin (Shanghai, China), Meryer (Shanghai, China), Energy Chemical Co. (Shanghai, China), and J&K (Beijing, China). LC-MS-grade formic acid was obtained from Aladdin (Shanghai, China). LC-MS-grade methanol (MeOH) and acetonitrile (ACN) were acquired from Merck (Darmstadt, Germany). Ultrapure water (H_2_O) was generated using a Milli-Q system (Millipore, Bedford, MA, USA).

### 2.3. Criteria for Staging Bunyavirus Patients

In accordance with the Diagnosis and Treatment Guidelines for Severe Fever with Thrombocytopenia Syndrome (2023 Edition) issued by the National Health Commission of the People’s Republic of China and the National Administration of Traditional Chinese Medicine [24], patients were classified to three clinical stages: the fever stage (A), the organ dysfunction stage (B), and the convalescence stage (C). A total of 177 specimens were collected from 78 patients across these different clinical stages.

During the fever state (A), characterized by an acute onset, patients typically present with fever (body temperature ranging from 38 to 40 °C), accompanied by symptoms such as fatigue, loss of appetite, nausea, and vomiting. Some cases may also exhibit muscle pain and diarrhea, while a few individuals may display apathy. Physical examination often reveals enlarged, tender, superficial lymph nodes, particularly in the unilateral groin, neck, and armpit area. In more severe cases, these lymph nodes may show marked local redness, swelling, warmth, and pain.

In the stage of multiple organ dysfunction (B), patients may exhibit persistent fever, multiple organ dysfunction, extreme fatigue, and worsening gastrointestinal symptoms. In some instances, this stage may also be characterized by involuntary shaking of the jaw and limbs, along with increased muscle tone. Severe cases may manifest as skin ecchymosis, gastrointestinal bleeding, pulmonary hemorrhage, irritability, delirium, convulsions, and coma. Some patients may succumb to complications, such as circulatory failure, respiratory failure, or hemorrhage.

During the convalescence stage (C), patients’ body temperature returns to normal, clinical symptoms gradually subside, and recovery typically occurs within approximately 2 weeks. However, the course of SFTS may be prolonged in patients with complications.

As shown in Figure S1, based on clinical presentations, the data were classified into 3 groups according to the SFTS stage from the onset of symptoms to outcomes: stage A (0–8 days), stage B (6–15 days), and stage C (12–21 days).

### 2.4. Serum Sample Collection and Preparation

The leftover blood samples were collected from patients at admission, during treatment, and before discharge. Serum samples were obtained after centrifugation at 1600× *g* for 10 min. The serum samples were divided into aliquots and preserved at −80 °C for subsequent analysis.

Each serum sample (100 µL) was mixed with 400 µL of pre-chilled MeOH for extraction and protein precipitation. The mixture was vortexed for 60 s, followed by incubation at −20 °C for 20 min. Subsequently, the samples were centrifuged at 10,000× *g* for 20 min at −4 °C. The supernatant was collected, dried under nitrogen, and dissolved in a mixture of 20 µL of internal standard (N-acyl glycine (C2–C23) standards) [25] and 130 µL of H_2_O. The mixture was vortexed for 3 min and then centrifuged at 10,000× *g* for 10 min for LC-MS analysis. Blank samples were also processed, replacing serum with 100 µL of H_2_O and following the same procedure.

To evaluate analytical precision, quality control (QC) samples were generated by combining equal volumes (30 µL) from each serum sample. Serum samples were analyzed in a randomized sequence, with QC samples injected every 10 runs throughout the experiment to monitor and ensure data accuracy and consistency.

### 2.5. LC-MS Analysis

Serum samples were analyzed using an ultra-high-performance liquid chromatography–quadrupole time-of-flight (UHPLC-Q-TOF) mass spectrometry system. The system consisted of an Agilent 1290 Infinity II liquid chromatography system, coupled with an Agilent 6546 Q-TOF mass spectrometer (Agilent Technologies, Palo Alto, CA, USA), equipped with an Agilent Jet Stream electrospray ionization (ESI, Turbo Ionspray) source. For liquid chromatography separation, an ACQUITY UPLC^®^ HSS T3 column (2.1 × 100 mm, 1.8 μm) was employed. The flow rate of the mobile phase was maintained at 0.4 mL/min, and the column temperature was set at 40 °C. The mobile phase included 0.1% formic acid in water (*v*/*v*, solvent A) and 0.1% formic acid in ACN (*v*/*v*, solvent B) for both positive and negative ion mode analyses. The gradient program was as follows: 0–1 min, 2% B; 1–23 min, 2–98% B; 23–25 min, 98% B; 25–25.1 min, 98–2% B; 25.1–30 min, 2% B.

Mass spectrometry analysis was performed in full-scan mode, with the mass-to-charge ratio (*m*/*z*) range set from 50 to 1000. The acquisition rate was 2.5 spectra per second. The electrospray ionization (ESI) parameters were configured as follows: ion transfer tube temperature, 320 °C; declustering potential, 120 V; spray voltage, 3500 V for positive ion mode and 3000 V for negative ion mode; sheath gas flow rate, 11 L/min; sheath gas temperature, 350 °C; drying gas flow rate, 8 L/min; nebulizer pressure, 35 psi.

For MS2 analysis, spectra of different metabolites were acquired using the auto-MS/MS mode. The MS scan rate was set at 3 spectra per second, while the MS/MS scan rate was 8 spectra per second. MS2 fragment ions were generated through collision-induced dissociation (CID), with collision energies of 10, 20, 30, and 45 eV. The intensity threshold was set to 10,000, with a maximum of 6 precursors analyzed per cycle. Dynamic exclusion was applied with a time of 0.2 min.

### 2.6. Data Processing

Raw data were acquired using Agilent 6546 MassHunter Workstation software (version 10.1, Agilent Technologies), and the resulting raw files (.d format) were converted to abf format using the ABF_Converter tool. Peak detection, deconvolution, alignment, blank subtraction, normalization, and additional processing were conducted with MS-DIAL software (version 4.70) to generate comprehensive feature lists. To refine the data, redundant ions, such as isotope peaks, adduct ions, duplicate peaks, and contaminant ions, were removed using the web-based MS-FLO tool [26].

To eliminate metabolites with a high proportion of missing values, the 80% rule was applied, retaining only metabolites present in at least 80% of the samples. Missing values were imputed using 1/5 of the minimum value observed among the remaining metabolites [27]. For metabolites detected on multiple platforms, the one with the smallest relative standard deviation (RSD) in QC samples was retained. Additionally, only metabolites with an RSD below 30% in QC samples were considered for further analysis. To approximate a normal distribution, logarithmic transformation was applied to the data prior to statistical analysis

Metabolite annotation was performed using MS2 spectra acquired in auto-MS/MS mode. The annotation process utilized the Global Natural Products Social Molecular Networking (GNPS; https://gnps.ucsd.edu/ProteoSAFe/static/gnps-splash.jsp access on 20 January 2025) and SIRIUS 4.9.15 (https://bio.informatik.uni-jena.de/sirius/ access on 20 January 2025). Significantly altered metabolites were annotated through standard confirmation, matching against the public MS2 database, and MS/MS interpretation. Annotation levels were classified according to the Metabolomics Standards Initiative (MSI) guidelines [28,29].

### 2.7. Statistical Analysis

Descriptive statistics were performed to analyze the demographic and clinical characteristics of the study population. Comparisons between the survival and fatal groups were conducted using chi-square tests for categorical variables and Wilcoxon rank-sum tests for continuous variables.

Multivariate statistical analyses, including principal component analysis (PCA) and partial least squares discriminant analysis (PLS-DA), were performed using SIMCA 14.1 (Umea, Sweden). Key classification variables were identified based on variable importance in projection (VIP) scores derived from the PLS-DA model. The reliability and accuracy of the PLS-DA model were assessed through 200 permutation tests.

Metabolic pathway analysis, single-factor analysis, and correlation analysis were performed using MetaboAnalyst 5.0 and IBM SPSS 20.0. Volcano plots and boxplots were visualized using Origin software (OriginPro 2024 (64-bit) SR1). Hierarchical clustering heatmaps, pathway analysis plots, and enrichment analysis plots were generated using R version 4.1.2 (R Foundation for Statistical Computing, Vienna, Austria). Lasso regression analysis was conducted in R to select metabolites for constructing a metabolic prognostic model, and model performance was assessed through receiver operating characteristic (ROC) curves plotted using the random forest algorithm.

The random forest model was developed using 20-fold cross-validation on the training set. To enhance interpretability and based on prior research, we selected five different machine learning models: logistic regression, ElasticNet linear regression, partial least squares discriminant analysis (PLS-DA), support vector machine (SVM), and random forest [21]. Hyperparameter tuning and feature selection for each model were optimized using 20-fold cross-validation combined with a grid search approach. Model performance was measured by the area under the receiver operating characteristic curve (AUC). Upon optimization, the random forest model demonstrated the highest AUC within the cross-validated training dataset.

## 3. Results

### 3.1. Clinical Features of Study Population

We first described the clinical indicators of the survival and fatal groups across the three stages (Table S2) to understand their characteristics at different stages of the disease. Due to the difficulty of collecting clinical data for fatal cases at stage C, information from this stage is not presented in the table. The analysis revealed that at stages A and B, fatal patients exhibited: (1) higher viral loads and (2) elevated levels of serum aspartate transaminase/alanine transaminase (AST/ALT), blood urea nitrogen (BUN), creatinine, lactate dehydrogenase (LDH), high-sensitivity troponin I (HSTNI), and activated partial thromboplastin time (APTT), indicating the presence of multi-organ damage in severe cases. Of note, the platelet was significantly decreased in fatal cases during stage A, but there was no difference during stage B. The reason for this phenomenon may be due to the infusion of exogenous platelets for treatment.

**Figure 2:**
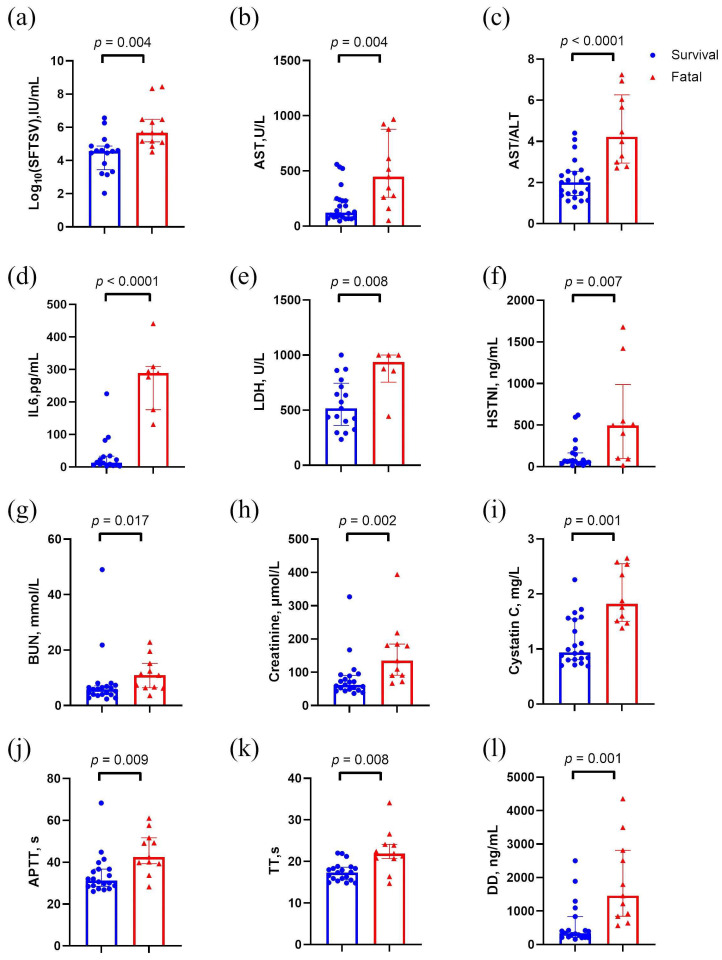
Clinical characteristics of stage B patients in the discovery set. (**a**) Comparison of SFTSV load between Survival and Fatal groups. (**b**,**c**) Comparison of liver function index between Survival and Fatal groups. (**d**) Comparison of IL6 between Survival and Fatal groups. (**e**,**f**) Comparison of cardiac function index between Survival and Fatal groups. (**g**–**i**) Comparison of renal function index between Survival and Fatal groups. (**j**–**l**) Comparison of coagulation function indicators between Survival and Fatal groups. The *p* values were calculated using a two-sided Mann–Whitney U test. Abbreviations: AST, aspartate aminotransferase; ALT/AST, aspartate transaminase/alanine transaminase; APTT, activated partial thromboplastin time; BUN, blood urea nitrogen; HSTNI, high-sensitivity troponin I; IL-6, interleukin-6; LDH, lactate dehydrogenase; TT, thrombin time.

As stage B represents a critical period of disease progression, we performed statistical analyses to further investigate differences at this stage. To account for potential confounding by age and sex, we compared their distributions between the groups and found no significant differences in age (*p* = 0.45) or sex (*p* = 0.25). As illustrated in Figure 2, fatal patients at stage B demonstrated the following notable features: (1) significantly higher levels of viral load, AST, interleukin-6 (IL-6), LDH, HSTNI, BUN, creatinine, and cystatin C, further underscoring the prevalence of multi-organ damage in severe cases (Figure 2a–i), and (2) prolonged activated partial thromboplastin time (APTT) and thrombin time (TT), as well as increased D-dimer (DD) concentrations, suggesting activation of the coagulation system and a hypercoagulable state during the progressive phase (Figure 2j–l). Similarly, in the test set, no significant differences were found in age (*p* = 0.07) or sex (*p* = 0.19), and the clinical features demonstrated comparable distribution patterns (Table S3).

**Figure 3:**
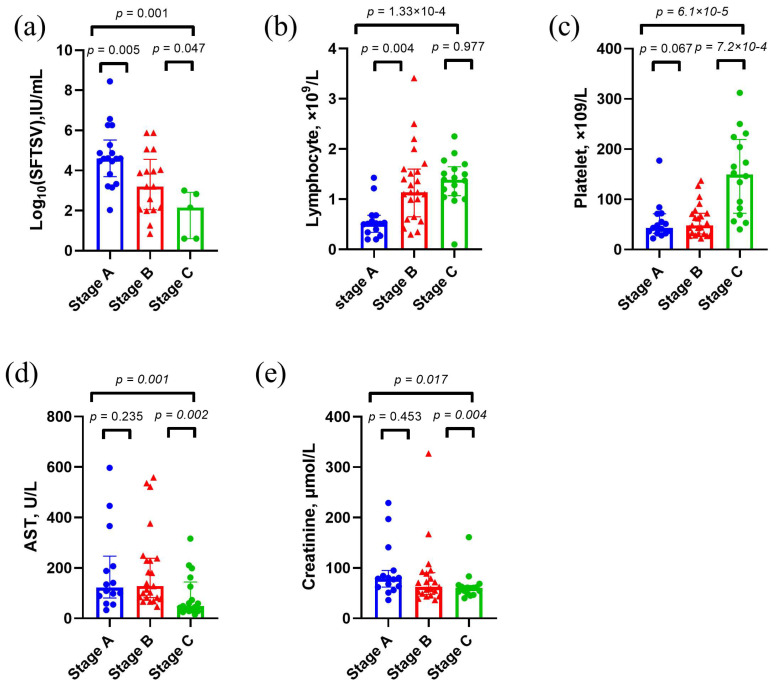
Clinical characteristics of survival cases in the discovery set. Comparison of SFTSV load (**a**), lymphocyte counts (**b**), platelet counts (**c**), AST (**d**) and (**e**) creatinine levels among three Stages are shown. *p* values were calculated using two-sided Mann–Whitney U test. Abbreviations: AST, aspartate aminotransferase.

**Figure 4:**
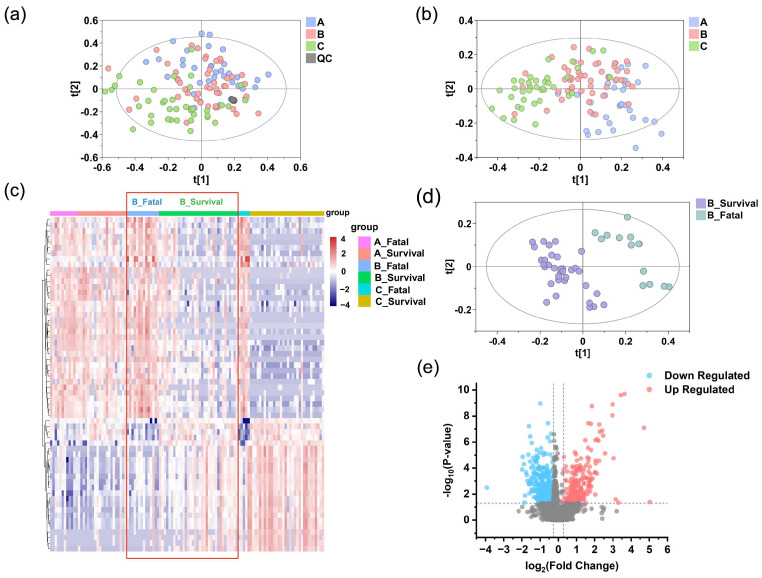
Reprogrammed serum metabolic landscape of SFTS patients. Identification of differential serum metabolomic profiles in SFTS patients at stages A (blue), B (pink), and C (green) in the discovery set. PCA (**a**) and PLS-DA (**b**) analyses indicate grouping patterns. The tight clustering of QC samples (gray points) in both positive and negative ion modes confirms the accuracy and reliability of the data. (**c**) Heatmap of serum metabolites in the fatal and survival groups at stages A, B, and C. Red denotes higher concentrations, while purple signifies lower concentrations, with more pronounced metabolite differences evident at stage B compared to stage A, as highlighted by the red box. (**d**) The PLS-DA model of serum metabolomics data distinguishes between the fatal (teal) and survival (purple) groups at stage B. (**e**) A volcano plot of metabolites detected in the fatal and survival groups at stage B shows significantly different metabolites in red (upregulated) and blue (downregulated), while others are shown in gray.

Based on the preceding analysis, we further investigated the dynamic changes in the survival group across different stages to reveal the trends in clinical characteristics during the recovery process (Figure 3). The findings revealed the following: (1) in the convalescence stage (stage C), viral load decreased significantly (Figure 3a), (2) lymphocyte counts were at their lowest in stage A, with over half of the cases below the normal range, followed by gradual recovery (Figure 3b), (3) platelet counts were at their lowest in stage A but increased significantly with disease improvement, resolving the platelet crises (Figure 3c), and (4) serum AST and creatinine levels were markedly elevated at stage A and significantly decreased by stage C (Figure 3d,e). In contrast, patients in the fatal group consistently exhibited high viral loads, severe thrombocytopenia, and persistent liver and kidney dysfunction throughout the disease course, with no apparent improvement.

### 3.2. Untargeted Metabolomics Analysis of Patients with Severe Fever with SFTS

An LC-MS-based untargeted metabolomics analysis was conducted on 118 serum samples collected from 52 patients in the discovery cohort with SFTS. This analysis identified 1255 metabolic features in positive ion mode and 1106 in negative ion mode. To assess the overall accuracy of the analysis, PCA was performed on the detected features. The PCA score plots demonstrated tight clustering of the QC samples in both ion modes (Figure 4a), indicating good reproducibility of the LC-MS analysis and confirming the accuracy and reliability of the obtained metabolomic data.

### 3.3. Exploratory Analysis of Grouping

Through the application of partial least squares discriminant analysis (PLS-DA) and permutation testing, we successfully visualized and validated the differences in metabolites among the three stages (stages A, B, and C). As shown in Figure 4b, the PLS-DA model indicates grouping patterns across the three stages. The robustness of the PLS-DA model was further confirmed by 200 permutation tests (Figure S2A), demonstrating no overfitting and good discriminative ability.

Furthermore, the samples from the three stages were divided into six independent groups based on patient outcomes (survival or fatal). The hierarchical clustering heatmap revealed minimal metabolic differences between survival and fatal groups at stage A, which became markedly pronounced at stage B and further intensified at stage C (Figure 4c). This pattern prompted us to hypothesize that survivors may begin showing signs of metabolic recovery as early as stage B, whereas fatal cases exhibit ongoing deterioration. The distinct metabolic profiles observed at stage B suggest it may represent a critical turning point for prognostic differentiation. Consequently, we sought to explore the metabolic changes at stage B, the distinctions between survival and fatal cases, and the potential of these differences as therapeutic targets. By applying the PLS-DA model to analyze the survival and fatal groups at stage B, a clear separation was observed (Figure 4d), and the model was validated as robust through 200 permutation tests (Figure S2B). Moreover, volcano plots revealed marked serum metabolic dysregulation in the fatal group (Figure 4e), with several metabolites exhibiting significant upregulation or downregulation trends (*p* < 0.05; FC > 1.2).

### 3.4. Significantly Altered Differential Metabolites

Based on the results from the exploratory analysis of the groups, we focused on the metabolic differences between the survival and fatal cases, examining how significant differential metabolites changed during disease progression. We selected differential metabolites between the survival and fatal groups at stage B. Applying the criteria of VIP values > 1.0 from the PLS-DA model and the multiple criteria of FC > 1.2 and *p* < 0.05 from the volcano plot, we identified 413 significantly altered metabolites. Specifically, 199 and 214 metabolites showed significant changes in positive and negative ion modes, respectively. Among the significantly changed metabolites, 170 were upregulated (53 from the positive ion mode and 117 from the negative ion mode), and 243 were downregulated (146 from the positive ion mode and 97 from the negative ion mode).

**Figure 5:**
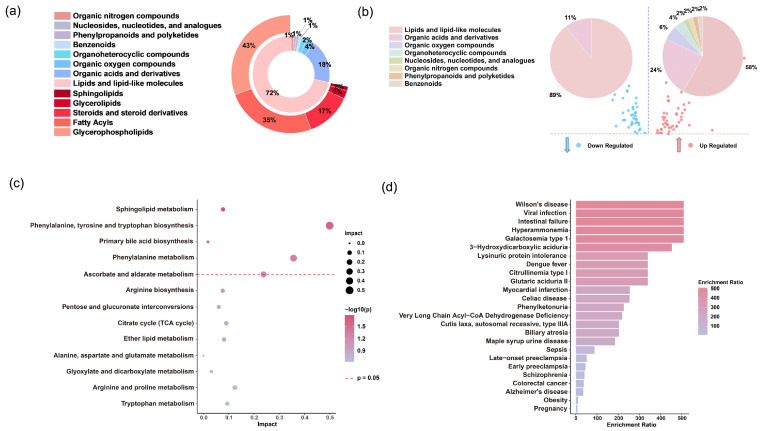
Analysis of differential serum metabolites. (**a**) Distribution of serum metabolites across super- and sub-classes in the discovery set. (**b**) Super-classes of metabolites significantly upregulated (red) and downregulated (blue) in the fatal group compared to the survival group. (**c**) Pathway analysis of significantly different metabolites between the fatal and survival groups according to the KEGG pathways. (**d**) Human disease states associated with SFTS-related metabolites based on previously published metabolomics data.

Then, 88 metabolites were annotated (Table S4), with 27 identified using standard compounds (level 1) and 54 matched against public databases based on MS2 spectra (level 2), and 7 metabolites were classified based on MS2 spectra data (level 3). Using the ClassyFire chemical classification system, these metabolites were categorized into seven super-classes: lipids and lipid-like molecules (72%), organic acids and derivatives (18%), organic oxygen compounds (4%), organoheterocyclic compounds (2%), benzenoids (1%), nucleosides, nucleotides, and analogues (1%), and phenylpropanoids and polyketides (1%), organic nitrogen compounds (1%). Notably, lipids and lipid-like molecules constituted the majority of the differential metabolites. Further subclassification of lipid molecules revealed glycerophospholipids (43%), fatty acyls (35%), steroids and steroid derivatives (17%), triglycerides (3%), and sphingolipids (2%). These findings indicate a greater and structurally diverse range of differential metabolites in fatal SFTS cases compared to those who recovered (Figure 5a).

Among the 88 annotated, significantly altered metabolites, 50 exhibited an upregulated trend, while 38 showed a downregulated trend. As shown in Figure 5b, lipids and lipid-like molecules were the top two super-classes for both upregulated (58%) and downregulated (89%) trends, followed by organic acids and derivatives (up 24% and down 11%). Hierarchical clustering analysis (Figure S3) revealed pronounced differences in the levels of annotated metabolites between the survival and fatal groups. Representative metabolites, such as lysophosphatidylcholine (LPC), showed a significant downregulation in the fatal group compared to the survival group, whereas bile acids and lysophosphatidylethanolamine (LPE) exhibited a noticeable upregulation trend (Figure S3). These changes may be related to the interaction mechanisms between SFTSV and the host.

To elucidate the relationship between these significantly altered metabolites and the metabolic state of SFTS patients, we utilized the Kyoto Encyclopedia of Genes and Genomes (KEGG) pathway database and MetaboAnalyst 5.0 for metabolic pathway analysis. This approach helped us investigate metabolites related to metabolic pathways and physiological changes in the patients’ serum. Our analysis revealed four major metabolic pathways that were significantly altered (*p* < 0.05): sphingolipid metabolism, the biosynthesis of phenylalanine, tyrosine, and tryptophan, primary bile acid biosynthesis, and phenylalanine metabolism (Figure 5c).

Additionally, the enrichment analysis indicated that several metabolites associated with SFTS are closely linked to the pathogenesis of various human diseases (Figure 5d), such as sepsis and dengue fever. This suggests that these metabolites likely participate in metabolic disruptions during disease states.

### 3.5. Selection and Evaluation of Potential Biomarkers

The significant changes in differential metabolites between the survival and fatal groups, as shown in the hierarchical clustering plot (Figure 4c), suggest the potential to establish a metabolic prognostic model. This model aims to predict disease severity from early metabolic profiles, assisting in clinical identification of severe cases and improving prognosis. We focused on the top 15 significantly different metabolites in the hierarchical clustering plot and further selected 8 potential biomarkers through lasso regression analysis and FDR testing. These included phenyllacetic acid, sphingosine-1-phosphate, isocitric acid, indole-3-lactic acid, phenylalanine, LysoPC(P-18:0/0:0), palmitoylcarnitine, and gluconic acid.

**Figure 6:**
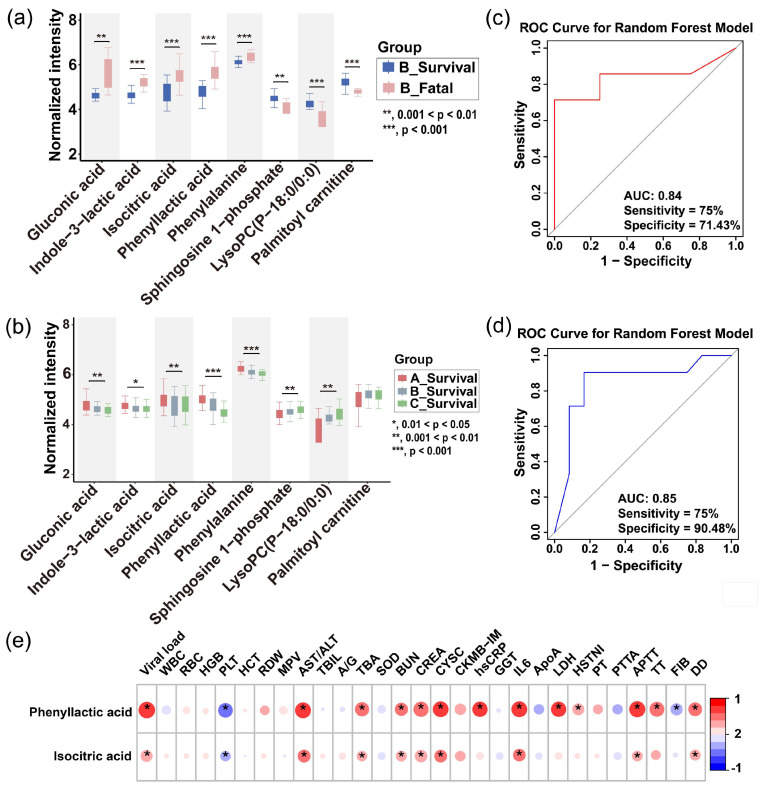
Prediction of disease severity in SFTS patients using machine learning. (**a**) Boxplots showing the intensities of potential biomarkers in the fatal and survival groups of SFTS patients (time point B). (**b**) Boxplots illustrating the longitudinal progression of potential biomarkers across disease stages (A, B, and C). Box limits represent the quartiles of each sample group. Receiver operating characteristic (ROC) curves of the metabolic prognostic model on the training (**c**) and testing (**d**) sets. (**e**) Heatmap of correlation coefficients between prognostic biomarkers and clinical parameters. The colors in the heatmap represent the positive (represented by red) or negative correlation (represented by blue). Abbreviations: WBC: white blood cell; RBC: red blood cell; HGB: hemoglobin; PLT: platelet; HCT: hematocrit; RDW: red cell distribution width; MPV: mean platelet volume; AST/ALT: aspartate transaminase/alanine transaminase; TBIL: total bilirubin; A/G: albumin/globulin ratio; TBA: total bile acid; SOD: superoxide dismutase; BUN: blood urea nitrogen; CREA: creatinine; CYSC: cystatin C; CKMB-IM: creatine kinase-MB isoenzyme; hsCRP: high-sensitivity C-reactive protein; GGT: gamma-glutamyl transferase; IL-6: interleukin-6; ApoA: apolipoprotein A; LDH: lactate dehydrogenase; HSTNI: high-sensitivity troponin I; PT: prothrombin time; PTTA: partial thromboplastin time activated; APTT: activated partial thromboplastin time; TT: thrombin time; FIB: fibrinogen; DD: D-dimer. *, 0.01 < *p* < 0.05; **, 0.001 < *p* < 0.01; ***, *p* < 0.001.

To further verify the correlation of these selected metabolites with SFTS pathogenesis and disease outcomes, we examined the concentration trends of these metabolites in the survival and fatal groups. We also explored the dynamic changes of these serum metabolites during disease progression in patients who ultimately recovered and were discharged. As shown in Figure 6a, phenyllactic acid, isocitric acid, indole-3-lactic acid, phenylalanine, and gluconic acid concentrations were significantly lower in the survival group compared to the fatal group, and they gradually decreased during recovery (Figure 6b). Conversely, sphingosine-1-phosphate, LysoPC(P-18:0/0:0), and palmitoylcarnitine concentrations were higher in the survival group (Figure 6a) and increased during the recovery process (Figure 6b). Additionally, at all three stages of disease progression, the concentrations of gluconic acid, indole-3-lactic acid, isocitric acid, phenyllactic acid, and phenylalanine remained lower in the survival group compared to the fatal group, while sphingosine-1-phosphate, LysoPC(P-18:0/0:0), and palmitoylcarnitine were consistently higher in the survival group (Figure S4). This consistent trend throughout the disease course indicates that the selected metabolites accurately reflect metabolic changes following SFTS infection and have significant predictive value.

To validate the reliability of potential biomarkers, we performed metabolomics analysis on serum samples from 26 SFTS patients in the test set. The results showed that the trends for seven metabolites—phenyllactic acid, isocitric acid, indole-3-lactic acid, phenylalanine, gluconic acid, sphingosine-1-phosphate, and LysoPC(P-18:0/0:0)—were consistent with the discovery set (Figure S5), while palmitoylcarnitine was not. Therefore, the former seven metabolites were retained. Subsequently, based on the metabolite profiles of the survival and fatal groups at stage B, we trained and optimized metabolic prognostic models using five machine learning approaches: logistic regression, ElasticNet linear regression, PLS-DA, SVM, and random forest. These models were then used to predict outcomes using data from stage A and validated on the test set. The AUC values for individual metabolites are presented in Table S5. Based on the combined performance of metabolites in the discovery and test sets, individual metabolites were selected and combined, as shown in Table S6. Our findings indicated that phenyllactic acid and isocitric acid performed best in cross-validation of the random forest model, achieving an AUC of 0.84, sensitivity of 75%, and specificity of 71% in the discovery set (Figure 6c), and an AUC of 0.85, sensitivity of 75%, and specificity of 90% in the test set (Figure 6d). This model outperformed other combinations and was more streamlined, and it was thus selected as the final prognostic model. According to multivariate regression analysis reported in the literature, older age, BUN levels, and APTT are independent risk factors for fatal outcomes [30]. A meta-analysis-derived model indicated that age, ATPP, Scr, BUN, hemorrhagic manifestations, and encephalopathy are critical factors for early assessment of mortality risk in patients with SFTS [31]. We conducted a comparative ROC analysis between these risk factors and the prognostic model. The metabolite model achieved an AUC of 0.85, outperforming age (AUC = 0.73), BUN levels (AUC = 0.65), and APTT (AUC = 0.78; Figure S6). Further analysis revealed that patients who eventually died had significantly higher scores using this model than those who recovered and were discharged, indicating its effectiveness in differentiating disease severity and predicting outcomes to some extent (Figure S7).

### 3.6. Correlation Analysis with Clinical Information

Previous studies have identified poor prognostic factors for SFTS, such as high serum viral RNA load, elevated levels of lactate dehydrogenase (LDH) and aspartate aminotransferase (AST), prolonged activated partial thromboplastin time (APTT), and increased interleukin-6 (IL-6) [3,32,33,34,35]. We assessed the correlation between phenyllactic acid and isocitric acid with these clinical indicators (Figure 6e). Phenyllactic acid concentration showed a significant positive correlation with viral load in patients (r = 0.71), suggesting that elevated phenyllactic acid levels might be associated with poor prognosis. Moreover, phenyllactic acid demonstrated significant positive correlations with several markers, including LDH (r = 0.60), IL-6 (r = 0.65), and high-sensitivity C-reactive protein (hs-CRP; r = 0.60), indicating a possible link between increased phenyllactic acid and myocardial damage, which is a critical factor in patient mortality due to cytokine storm [36]. Further analysis indicated significant correlations between phenyllactic acid and coagulation indicators (Table S7), such as positive correlations with APTT (r = 0.67), thrombin time (TT, r = 0.57), and D-dimer (DD, r = 0.47), and a negative correlation with platelet count (PLT, r = −0.55). This suggests that impaired coagulation function and thrombocytopenia may be associated with elevated phenyllactic acid levels, indicating a potential link with coagulation abnormalities. Based on these associations, it can be speculated that high phenyllactic acid concentrations might correlate with clinical deterioration. Data also showed (Figure 6a,b) that phenyllactic acid levels were significantly higher in the fatal group than in the survival group, with a gradual decrease observed during the recovery process.

Metabolomics studies have shown that the tricarboxylic acid (TCA) cycle is notably affected in patients who develop sepsis following trauma compared to healthy controls, with significant changes in intermediate metabolites, particularly isocitric acid [37], suggesting interference in energy metabolism. Normal liver and kidney function heavily relies on balanced energy metabolism. Thus, disruption of the TCA cycle may be linked to impaired hepatic and renal functions. Our analysis further demonstrated significant positive correlations between isocitric acid concentrations and hepatic and renal function indicators, including the AST/ALT ratio (r = 0.41), serum creatinine (CREA, r = 0.39), and cystatin C (CYSC, r = 0.40). As liver and kidney function indicators deteriorated, isocitric acid concentrations increased, indicating that elevated isocitric acid may be closely related to liver and kidney impairment. Data also showed (Figure 6a,b) that isocitric acid levels were significantly higher in the fatal group than in the survival group, with a gradual declined observed during the recovery process.

These results suggest that the elevated levels of our predictor metabolites, phenyllactic acid and isocitric acid, were associated with poor prognosis in SFTS patients. Consequently, these two metabolites can distinguish between patients who recover and those who experience fatal outcomes.

## 4. Discussion

Our study aimed to analyze the impact of SFTSV on host metabolism from a metabolomics perspective, focusing on clinical outcomes and the progression of the disease. By comparing patients who succumbed to the disease with those who improved, we identified potential abnormal metabolic pathways. These pathways provide insights into the pathophysiological changes and mechanisms of SFTS, which could lead to the identification of potential therapeutic targets. Metabolites that exhibit significant changes during disease progression may reflect patient status and could be used to construct metabolic prognostic models for disease outcomes.

Metabolomics analysis of 52 SFTS patients revealed significant differences between the survival and fatal groups, identifying four key differential metabolic pathways: sphingolipid metabolism, the biosynthesis of phenylalanine, tyrosine, and tryptophan, primary bile acid biosynthesis, and phenylalanine metabolism, all of which experience severe metabolic disruptions.

Sphingolipid metabolism emerges as an important pathway in distinguishing between survival and fatal SFTS patients, especially given the significant decrease in sphingosine-1-phosphate (S1P) and notable increase in D-sphingosine observed in fatal cases. S1P is essential for regulating vascular function [38], and its decreased levels may indicate a disruption in the balance of vascular homeostasis. Endothelial dysfunction induced by viral infection has been strongly linked to a higher risk of mortality, especially in patients experiencing severe hemorrhagic complications [39]. The reduction of S1P in fatal cases suggests its involvement in a vicious cycle of endothelial dysfunction, a phenomenon also observed in diseases like sepsis and dengue fever [40]. Additionally, the strong positive correlation (r = 0.68) between S1P and high-density lipoprotein (HDL) suggests that HDL levels may partially reflect S1P levels and disease status. Furthermore, in patients who eventually recovered, we observed a gradual rebound in S1P levels. Therefore, therapeutic strategies aimed at increasing circulating S1P levels or enhancing signaling may represent new options for stabilizing patient conditions.

Phenylalanine metabolism is a critical metabolic pathway in SFTS disease [23]. Our data indicated that levels of phenylalanine, phenyllactic acid, and hydroxyphenylacetic acid were significantly elevated in fatal cases, while they gradually declined in those who recovered. Phenylalanine is primarily metabolized in the liver, and inflammation or liver dysfunction may reduce its metabolic rate, leading to elevated phenylalanine levels in the body. Clinical data support this hypothesis, as liver function in fatal cases was significantly worse than in those who recovered, and metabolites of phenylalanine showed significant correlations with liver function. Additionally, impaired liver function could hinder the conversion of phenylalanine to tyrosine [41], resulting in the accumulation of phenyllactic acid and hydroxyphenylacetic acid. Similar phenomena have been observed in other infectious diseases, such as COVID-19 and sepsis, where elevated phenylalanine levels are closely related to disease severity [42,43]. Thus, the phenylalanine metabolic pathway may be involved in SFTSV invasion and lethal mechanisms.

Bile acid metabolism pathways exhibited significant dysregulation in SFTS patients. Bile acids, essential steroid compounds synthesized by the liver, were found at higher concentrations in fatal cases compared to those who recovered (Figure S3), with levels gradually decreasing as the patients improved. The liver, as one of the primary sites for SFTSV replication [44], becomes impaired, leading to disruptions in bile secretion and excretion. Concurrently, systemic inflammation might damage the biliary system, causing cholestasis and elevated serum bile acid levels. The higher bile acid levels in fatal cases compared to those who recovered were associated with worsening liver dysfunction, supporting this hypothesis.

Previous literature has suggested that certain bile acids, such as chenodeoxycholic acid (CDCA), glycochenodeoxycholic acid (GCDCA), taurochendeoxycholic acid (TCDCA), and taurodeoxycholic acid (TDCA), are significantly elevated in survival cases and may exert protective effects by inhibiting inflammatory responses [45]. However, our data differed from some studies [46,47], showing that bile acids continued to increase in fatal cases, a phenomenon also observed in severe COVID-19 cases, where elevated bile acids correlate with disease severity. We speculated that bile acids may have anti-inflammatory effects in the early stages of the disease, but as the disease progresses, damage to the liver and biliary system impedes normal bile metabolism, leading to bile acid accumulation in the blood and further exacerbating hepatocellular damage.

Receptors for bile acids, namely, FXR and TGR5, have recently gained considerable attention in the scientific community [48,49]. FXR not only regulates metabolic balance and maintains gut barrier integrity but also directly modulates the immune system [50]. Clinically, drugs like obeticholic acid (OCA) have been used to activate FXR, reducing bile acid synthesis and mitigating excessive immune responses [51]. Controlling the abnormal elevation of bile acids might aid in the recovery of SFTS patients. The significant differences in bile acid levels among patients with different outcomes suggest that studying bile-acid-related signaling pathways could provide new therapeutic targets for SFTS.

Lipid metabolism plays a crucial role in the progression of SFTS. Studies show that various lysophosphatidylcholines (LPCs) tend to decrease in fatal cases while gradually increasing in recovering patients; conversely, lysophosphatidylethanolamines (LPEs) rise in fatal cases and decrease in those who recover (Figure S3). LPC, an important phospholipid derivative, is associated with the progression of various diseases, including sepsis and liver cirrhosis, and is closely linked to increased mortality risk [52,53]. LPE, on the other hand, tends to elevate in inflammatory, metabolic, and infectious diseases. LPC is primarily derived from phosphatidylcholine (PC), while PC derives from phosphatidylethanolamine (PE) [54]. Therefore, it is hypothesized that more PE is converted to LPE, resulting in decreased PC and, consequently, reduced LPC and increased LPE levels.

LPC plays a crucial role in combating viral infections by activating macrophages, promoting the phagocytosis of viral particles and infected cells [55], and exhibiting anti-platelet aggregation properties. Studies have shown that LPC regulates platelet aggregation via serum phospholipase and exhibits a dose-dependent inhibitory effect [56]. This anti-platelet aggregation function is particularly important in SFTS patients. Previous literature has suggested that SFTS patients suffer from arginine deficiency, which affects the L-arginine/nitric oxide synthase/nitric oxide pathway in platelets, leading to their excessive activation and reduction [22]. As LPC levels decrease, its anti-platelet aggregation effect weakens, making patients more susceptible to abnormal platelet activation due to arginine deficiency, potentially leading to adverse outcomes. In patients who show improvement, the rise in LPC levels may help restore this anti-platelet function. Therefore, supplementing LPC might represent a novel therapeutic strategy to improve the prognosis of SFTS patients.

The tryptophan metabolism pathway is closely implicated in the pathophysiology of SFTS. Prior studies have highlighted a significant elevation of kynurenine, a critical metabolite derived from tryptophan, in the urine of SFTS patients compared to healthy controls, with markedly higher concentrations observed in fatal cases relative to survivors [23]. These changes may lead to excessive cytokine levels, potentially initiating a cytokine storm that increases the risk of mortality in patients. Consistent with the role of kynurenine in differentiating disease severity across viral infections, the study by Liu et al. showed that kynurenine was able to distinguish dengue hemorrhagic fever from milder dengue fever [19]. Our findings also demonstrated that serum kynurenine levels were significantly higher in fatal cases compared to those who recovered, and longitudinal analysis showed that kynurenine levels gradually decreased in improving patients, consistent with disease stabilization. The variation in kynurenine levels may provide a potential biomarker for prognostic assessment in SFTS and further supports the critical role of the tryptophan metabolism pathway in disease progression and lethal mechanisms.

Based on significant differences in metabolite profiles, we developed a prognostic model for the severity of SFTS. Through rigorous screening, phenyllactic acid and isocitric acid were identified as key predictor metabolites, demonstrating an impressive area under the curve (AUC) of 0.85 in a random forest model applied to the test set. This model exhibited a sensitivity of 75% and specificity of 90% (Figure 6c), surpassing other combinations of metabolites. These two metabolites were found to exhibit abnormal elevation in SFTS patients, with a more pronounced effect observed in those who succumbed (Figure 6a), indicating a close association with the metabolic state following viral infection. Due to their ease of detection and strong potential for clinical application, phenyllactic acid and isocitric acid serve as valuable references for early intervention and prognostic evaluation.

## 5. Conclusions

In summary, this study utilized serum to reveal the comprehensive metabolic profile of SFTS patients. By comparing longitudinal and cross-sectional data, we explored the metabolic alterations in fatal SFTS cases and constructed a metabolic prognostic model. Our findings indicated significant alterations in several metabolic pathways in fatal cases, including sphingolipid metabolism, biosynthesis of phenylalanine, tyrosine, and tryptophan, primary bile acid biosynthesis, and phenylalanine metabolism. These significant metabolic changes may offer new targets for the diagnosis and treatment of SFTS, such as S1P, LPC, and bile acids. The metabolic prognostic model could aid in the early clinical diagnosis of severe cases, facilitating rapid and effective treatment.

## Data Availability

All data that support the findings of the study are within the manuscript or in the Supplementary Materials.

## Supplementary Materials

The following supporting information can be downloaded at: https://www.mdpi.com/article/10.3390/metabo15040228/s1. Table S1: Other common viral infections in the discovery set. Table S2: Clinical characteristics of SFTS patients in the discovery set. Table S3: Clinical characteristics of SFTS patients in the test set. Table S4: Annotated significantly different metabolites in the serum of SFTS patients. Table S5: The performance of single biomarkers. Table S6: The performance of the biomarker combination models. Table S7: Associations between prognostic biomarkers and patient parameters. Figure S1: Duration of illness for each stage (A, B, and C). Figure S2: Cross-validation plot, with a permutation test repeated 200 times, of the PLS-DA score plot. Figure S3: A hierarchical clustering heatmap of 88 annotated differential serum metabolites, comparing the fatal and survival groups in SFTS patients. Figure S4: Comparison of metabolic profiles at three stages of disease progression in SFTS patients. Figure S5: The trends of potential biomarkers in the test set of patients. Figure S6: ROC curves for age, BUN, and APTT. Figure S7: The patient scores derived from the metabolic prognostic model output.
