# Supplementary material for: Metabolic Profiling Reveals Potential Prognostic Biomarkers for SFTS: Insights into Disease Severity and Clinical Outcomes
Source: Metabolites. 2025 Mar 27;15(4):228. doi: 10.3390/metabo15040228 (PMC12028903; doi:10.3390/metabo15040228)

Supplementary Material

for

Metabolic Profiling Reveals Potential Prognostic Biomarkers for SFTS: Insights into Disease Severity and Clinical Outcomes

Zhuo-Min Zhu[1,2#], Huan-Yu Liu[3, 4 #], Na An[1,2#], An-Ling Li[3,#], Jia Li[1,2], Sai-Jun Wang[2], Gui Yang[3], Yong-Wei Duan[3], Ying Yang[3], Mei Zhang[7], Quan-Fei Zhu[1*], Song-Mei Liu[3,5*], Yu-Qi Feng[1,2,6]

[1] School of Bioengineering and Health, Wuhan Textile University, Wuhan 430200, China

[2] School of Public Health, Wuhan University, Wuhan 430072, China

[3] Department of Clinical Laboratory, Center for Gene Diagnosis & Program of Clinical Laboratory, Zhongnan Hospital of Wuhan University, Wuhan 430071, Hubei, China

[4] Department of Obstetrics, Zhongnan Hospital of Wuhan University, Wuhan 430071, Hubei, China.

[5] Hubei Province Key Laboratory of Allergy and Immunology, School of Basic Medical Sciences, Wuhan University, Wuhan 430071, Hubei, China.

[6] Frontier Science Center for Immunology and Metabolism, Wuhan University, Wuhan 430071, China

[7] Department of Clinical Laboratory, Ezhou Hospital of Traditional Chinese Medicine, Ezhou 436000, Hubei, China.

[#] These authors contributed equally to this work.

* Corresponding authors: Quan-Fei Zhu. E-mail: qf_zhu@whu.edu.cn; Song-Mei Liu. E-mail: smliu@whu.edu.cn

The supporting information includes following items:

Page S3	Table S1 Other common viral infection in the discovery set.
Page S4-5	Table S2 Clinical characteristics of SFTS patients in discovery set.
Page S6	Table S3 Clinical characteristics of SFTS patients in test set.
Page S7-15	Table S4 Annotated significantly different metabolites in the serum of SFTS patients.
Page S16	Table S5 The performance of single biomarkers.
Page S17	Table S6 The performance of the biomarker combination models.
Page S18	Table S7 Associations between prognostic biomarkers and patient parameters.
Page S19	Figure S1 Duration of illness for each stage (A, B, C).
Page S20	Figure S2 Cross-validation plot with a permutation test repeated 200 times of the PLS-DA score plot.
Page S21	Figure S3 A hierarchical clustering heatmap of 88 annotated differential serum metabolites comparing the fatal and survival groups in SFTS patients.
Page S22	Figure S4 Comparison of metabolic profile at three stages of disease progression in SFTS patients.
Page S23	Figure S5 The trends of potential biomarkers in the test set of patients.
Page S24	Figure S6 ROC curves for Age, BUN, and APTT.
Page S25	Figure S7 The patient scores derived from the metabolic prognostic model output.

Table S1 Other common viral infection in the discovery set. The table presents information on other common viral infections in the discovery set, including Hepatitis B virus (HBV), Epstein-Barr virus (EBV), Influenza A virus (FluA), and Influenza B virus (FluB).

Variables	Total (%)	Survival (%)	Fatal (%)	$\chi2$	p
HBV positive	7 (13.5%)	5 (13.5%)	2 (13.3%)	0.032	0.986
EBV positive	3 (5.8%)	2 (5.4%)	1 (5.9%)	0.031	0.860
FluA positive	0 (0 %)	0 (0 %)	0 (0 %)	NA	NA
FluB positive	1 (1.9 %)	0 (0 %)	1 (6.7%)	2.515	0.288

Abbreviation: HBV, hepatitis B virus; EBV, Epstein-barr virus; FluA, influenza A virus; FluB, influenza B virus.

Table S2 Clinical characteristics of SFTS patients in discovery set. Due to the difficulty of collecting clinical data for deceased patients at stage C, information from this stage is not presented in the table.

Characteristic	Stage A		Stage B		Stage C
	Survival（n=21）	Fatal(n=12)	Survival(n=34)	Fatal(n=14)	Survival(n=32)
Age (year)	59.50 (50.75, 68.25)	68.00 (66.00, 70.00)	64.00 (58.00, 69.50)	68.00 (64.50, 73.75)	62.00 (59.00, 62.00)
Sex (Male%)	25.00	22.22	21.74	40.00	29.42
Viral load	4.57 (3.70, 4.87)	5.67 (5.16, 6.48)	2.89 (2.04, 3.96)	5.36 (4.69, 6.30)	2.49 (1.76, 2.88)
WBC ($\times10^9$/L)	2.44 (1.56, 4.01)	2.99 (1.74, 3.72)	3.27 (2.29, 5.84)	3.38 (1.81, 4.88)	4.10 (3.42, 4.95)
RBC ($\times10^{12}$/L)	4.01 (3.83, 4.27)	2.99 (1.74, 3.72)	3.93 (3.75, 4.50)	3.86 (3.67,4.63)	3.68 (3.33,3.90)
HGB (g/L)	120.50 (111.00, 127.25)	116.00 (104.00, 127.00)	118.00 (109.00, 127.00)	112.00 (87.75, 136.25)	109.00 (102.40, 116.00)
PLT ($\times10^9$/L)	45.50 (35.00, 71.25)	34.00 (34.00, 41.00)	48.00 (31.00, 71.50)	38.00 (34.75, 39.75)	152.00 (82.00, 224.00)
HCT (%)	36.40 (31.95, 37.55)	36.30 (31.90, 36.60)	34.80 (32.80, 37.95)	32.10 (24.35, 37.33)	32.90 (30.50, 34.50)
RDW (%)	13.35 (12.67, 14.00)	14.30 (13.20, 15.30)	13.30 (13.00 13.85)	14.20 (13.45, 15.73)	13.40 (12.90, 13.90)
MPV (fL)	11.60 (10.50, 14.15)	10.6 (9.45, 12.58)	10.75 (9.78, 12.20)	9,60 (9.25,10.93)	9.75 (9.23, 11.53)
AST/ALT	2.49 (2.00, 3.08)	4.46 (2.76, 5.33)	2.00 (1.42, 2.50)	4.23 (3.08, 5.95)	0.83 (0.57, 1.56)
AST (μ/L)	138.00 (95.75, 336.00)	317.00 (203.00, 1042.00)	121.00 (82.00, 235.75)	397.00 (264.50, 787.25)	49.00 (32.00, 126.00)
TBIL (μmol/L)	8.70 (5.77, 10.35)	7.70 (6.70,14.10)	7.80 (7.25, 16.80)	11.90 (10.32, 15.23)	15.50 (13.00, 23.00)
A/G	1.24 (1.11, 1.30)	2.99 (1.74, 3.72)	1.07 (0.96, 1.22)	1.13 (1.00)	1.07 (0.91, 1.30)
TBA (μmol/L)	6.60 (5.25, 11.40)	13.8 (10.5, 40.7)	3.40 (2.50, 4.65)	15.40 (2.78, 27.38)	2.70 (1.90, 4.00)
SOD (U/mL)	113.00 (110.40, 134.00)	108 (106.25, 123.25)	110.30 (98.18, 126.00)	110.55 (107.83, 118.15)	147.05 (132.40, 151.75)
BUN (mmol/L)	4.95 (3.69, 6.76)	6.60 (5.10, 9.84)	5.89 (4.00, 6.90)	9.15 (6.47, 12.15)	4.75 (3.36, 5.38)
CREA (μmol/L)	79.60 (63.40, 96.45)	75.20 (63.90, 132.70)	62.40 (49.10, 89.00)	122.20 (91.10, 180.75)	60.50(52.00,63.83)
CYSC (mg/L)	1.11 (1.00, 1.36)	2.13 (1.20, 2.26)	0.94 (0.81, 1.53)	1.82 (1.53, 2.49)	1.00 (0.86, 1.14)
CKMB-IM (U/L)	30.00 (21.50, 45.50)	59.00 (48.25, 67.50)	23.00 (18.00, 36.00)	56.00 (30.60, 58.50)	15.00 (11.00, 39.00)
hsCRP (mg/L)	1.04 (0.22, 5.86)	1.27 (1.21, 4.12)	1.29 (0.64, 17.83)	33.21 (25.98, 39.95)	1.00 (0.87, 4.33)
GGT (U/L)	20.00 (15.25, 36.25)	55.00 (44.00, 178.00)	57.00 (24.00, 145.50)	50.00 (26.00, 144.25)	84.00 (40.00, 132.00)

Characteristic	Stage A		Stage B		Stage C
	Survival	Fatal	Survival	Fatal	Survival
IL6 (pg/mL)	39.00 (14.10, 67.30)	91.65 (44.30, 122.25)	13.20 (7.09, 31.92)	291.50 (251.75, 342.00)	6.42 (3.65, 16.20)
ApoA (g/L)	0.88 (0.69, 0.10)	0.80 (0.78, 0.92)	0.94 (0.81, 1.01)	0.83 (0.80, 0.88)	0.86 (078, 0.97)
LDH (U/L)	732.00 (390.50, 996.50)	789.50 (639.75, 1000.00)	545.00 (405.25, 759.50)	938.00 (861.75, 1000.00)	317.00 (253.00,459.00)
HSTNI (ng/mL)	71.25 (25.90, 149.68)	91.65 (44.30, 122.25)	67.30 (44.65, 157.50)	496.80 (101.90, 551.40)	70.60 (50.83, 110.50)
PT (s)	11.60 (10.75, 11.95)	11.75 (11.28,12.70)	10.80 (10.35, 11.85)	10.80 (10.35, 11.85)	11.00 (10.90, 11.30)
PTTA (s)	94.00 (85.00, 107.50)	90.50 (78.50, 95.25)	102.50 (93.25, 112.75)	102.00 (88.25, 109.75)	31.65 (29.63, 33.13)
APTT (s)	38.00 (0.69, 1.00)	46.90 (43.65, 54.20)	32.00 (28.70, 37.46)	45.80 (39.43, 51.10)	31.65 (29.63, 33.13)
TT (s)	18.50 (17.65, 21.15)	20.20 (19.30, 30.70)	17.65 (15.90, 18.73)	21.60 (20.73, 24.05)	17.50 (17.20, 18.30)
FIB (g/L)	221.00 (185.50, 240.00)	192.50 (175.75, 264.00)	271.50 (248.75, 312.50)	248.50 (220.00, 288.25)	250.00 (209.25, 280.50)
DD (μg/mL)	1220.00 (733.00, 2255.00)	2801.00 (1224.25, 4136.75)	364.50 (242.75, 731.50)	1342.00 (864.75, 2311.25)	464.50 (368.25, 691.00)

All variables are presented in the form of IQR (Q1, Q3). **Viral load values** in the table have been logarithmically transformed to better to better reflect the distribution of the data.

Abbreviations: WBC: white blood cell; RBC: red blood cell; HGB: hemoglobin; PLT: platelet; HCT: hematocrit; RDW: red cell distribution width; MPV: mean platelet volume; AST/ALT: aspartate transaminase / alanine transaminase; TBIL: total bilirubin; A/G: albumin/globulin ratio; TBA: total bile acid; SOD: superoxide dismutase; BUN: blood urea nitrogen; CREA: creatinine; CYSC: cystatin C; CKMB-IM: creatine kinase-MB Isoenzyme; hsCRP: high-sensitivity C-reactive protein; GGT: gamma-glutamyl transferase; IL6: interleukin; ApoA: apolipoprotein A; LDH: lactate dehydrogenase; HSTNI: high-sensitivity troponin I; PT: prothrombin time; PTTA: partial thromboplastin time activated; APTT: activated partial thromboplastin time; TT: thrombin Time; FIB: fibrinogen; DD: D-dimer.

Table S3 Clinical characteristics of SFTS patients at stage B in the test set.

Characteristics	Stage B	
	**Survival**	**Fatal**
Age (year)	68.00 (60.50, 70.50)	76.00 (66.50, 78.00)
Sex (Male%)	42.11	71.43
Viral load	4.65 (3.76, 5.10)	5.28 (4.59, 5.53)
WBC (×10^9/L)	2.04 (1.48, 3.73)	5.00 (2.86, 6.18)
RBC (×10^{12}/L)	4.25 (3.91, 4.60)	4.34 (4.20, 4.88)
HGB (g/L)	125.00 (120.00, 136.35)	129.00 (122.50, 146.00)
PLT (×10^9/L)	52.00 (32.50, 61.00)	42.00 (24.00, 65.00)
HCT (%)	37.80 (35.40, 40.80)	38.50 (37.55, 44.25)
RDW (%)	13.30 (12.75, 13.90)	13.80 (13.15, 14.65)
MPV (fL)	10.75 (9.98, 11.65)	12.80 (12.50, 13.70)
AST/ALT	3.48 (2.30, 3.71)	3.11 (3.02, 3.60)
AST (μ/L)	204.00 (127.00, 489.50)	427.00 (266.00, 596.50)
TBIL (μmol/L)	8.90 (6.60, 11.90)	9.90 (8.70, 12.45)
A/G	1.20 (1.07, 1.33)	1.07 (0.97, 1.31)
TBA (μmol/L)	8.50 (5.05, 12.00)	6.70 (2.75, 28.30)
SOD (U/mL)	127.00 (109.75, 157.45)	121.00 (94.98, 141.00)
BUN (mmol/L)	5.10 (3.20, 8.20)	8.10 (7.56, 13.62)
CREA (μmol/L)	68.50 (62.25, 97.00)	100.10 (96.20, 112.85)
CYSC (mg/L)	1.19 (0.94, 1.75)	1.78 (1.65, 1.86)
CKMB-IM (U/L)	2.60 (1.50, 8.70)	9.55 (6.50, 10.20)
hsCRP (mg/L)	31.81 (1.73, 227.25)	7.33 (5.90, 85.60)
GGT (U/L)	28.00 (20.50, 63.50)	88.00 (50.00, 209.00)
IL6 (pg/mL)	50.80 (20.30, 117.00)	64.60 (49.30, 74.00)
ApoA (g/L)	0.99 (0.91, 1.02)	0.75 (0.69, 0.81)
LDH (U/L)	793.00 (555.00, 1000.00)	1000.00 (643.00, 1517.00)
HSTNI (ng/mL)	114.50 (80.92, 195.68)	170.60 (98.05, 512.80)
PT (s)	11.00 (10.55, 11.55)	11.80 (11.25, 12.25)
PTTA (s)	101.00 (96.00, 110.50)	90.00 (87.00, 99.00)
APTT (s)	35.90 (34.20, 39.70)	39.50 (36.45, 44.15)
TT (s)	18.20 (16.35, 20.25)	19.70 (17.10, 23.20)
FIB (g/L)	253.00 (201.50, 348)	245.00 (180.00, 304.00)
DD (μg/mL)	1321.00 (730.00, 2069.50)	2692.00 (1151.00, 3317.00)

All variables are presented in the form of IQR (Q1, Q3). **Viral load values** in the table have been logarithmically transformed to better to better reflect the distribution of the data.

Abbreviations: WBC: white blood cell; RBC: red blood cell; HGB: hemoglobin; PLT: platelet; HCT: hematocrit; RDW: red cell distribution width; MPV: mean platelet volume; AST/ALT: aspartate transaminase / alanine transaminase; TBIL: total bilirubin; A/G: albumin/globulin ratio; TBA: total bile acid; SOD: superoxide dismutase; BUN: blood urea nitrogen; CREA: creatinine; CYSC: cystatin C; CKMB-IM: creatine kinase-MB Isoenzyme; hsCRP: high-sensitivity C-reactive protein; GGT: gamma-glutamyl transferase; IL6: interleukin; ApoA: apolipoprotein A; LDH: lactate dehydrogenase; HSTNI: high-sensitivity troponin I; PT: prothrombin time; PTTA: partial thromboplastin time activated; APTT: activated partial thromboplastin time; TT: thrombin Time; FIB: fibrinogen; DD: D-dimer.

Table S4 Annotated significantly different metabolites in the serum of SFTS patients.

Name	*m/z*	RT	Mode	VIP	FC	*P*-value	FDR	Formula	HMDB ID	Subclass	Superclass	Level
Isocitric acid	191.0205	0.95	T3-ESI(−)	4.7	8.18	1.70E-05	7.72E-04	C6H8O7	HMDB0000193	Tricarboxylic acids and derivatives	Organic acids and derivatives	1
Sphingosine-1-phosphate	378.2416	14.15	T3-ESI(−)	2.2	0.44	1.09E-05	5.96E-04	C18H38NO5P	HMDB0000277	Phosphosphingolipids	Lipids and lipid-like molecules	1
3-Hydroxyoctanoic acid	159.1028	8.45	T3-ESI(−)	1.5	1.73	1.24E-03	1.47E-02	C8H16O3	HMDB0001954	Medium-chain hydroxy acids and derivatives	Organic acids and derivatives	1
Indole-3-lactic acid	204.0670	6.79	T3-ESI(−)	2.4	3.59	1.72E-09	8.11E-07	C11H11NO3	HMDB0000671	Indolyl carboxylic acids and derivatives	Organoheterocyclic compounds	1
lysoPC(P-18:0/0:0)	508.3763	18.67	T3-ESI(+)	3.1	3.12	9.07E-06	5.22E-04	C26H54NO6P	HMDB0013122	Glycerophosphocholines	Lipids and lipid-like molecules	1
2-Hydroxy-2-methylbutyric acid	117.0559	3.33	T3-ESI(−)	1.3	2.01	1.51E-04	3.50E-03	C5H10O3	HMDB0001987	Fatty acids and conjugates	Lipids and lipid-like molecules	1
Suberic acid	173.0821	6.33	T3-ESI(−)	1.6	1.91	5.00E-03	3.63E-02	C8H14O4	HMDB0000893	Fatty acids and conjugates	Lipids and lipid-like molecules	1
Sebacic acid	201.1135	8.80	T3-ESI(−)	1.3	1.69	9.04E-03	5.31E-02	C10H18O4	HMDB0000792	Fatty acids and conjugates	Lipids and lipid-like molecules	1
Hexadecanedioic acid	285.2074	15.10	T3-ESI(−)	1.5	2.65	1.37E-03	1.55E-02	C16H30O4	HMDB0000672	Fatty acids and conjugates	Lipids and lipid-like molecules	1
Palmitoyl carnitine	400.3424	15.68	T3-ESI(+)	1.9	2.43	2.57E-05	1.01E-03	C23H46NO4	HMDB0000222	Fatty acid esters	Lipids and lipid-like molecules	1
L-kynurenine	209.0934	2.77	T3-ESI(+)	2.3	0.36	7.09E-07	7.61E-05	C10H12N2O3	HMDB0000684	Carbonyl compounds	Organic oxygen compounds	1

Table S4 Annotated significantly different metabolites in the serum of SFTS patients (continued).

Name	*m/z*	RT	Mode	VIP	FC	*P*-value	FDR	Formula	HMDB ID	Subclass	Superclass	Level
Taurochenodesoxycholic acid	498.2909	11.67	T3-ESI(−)	2.8	3.13	2.46E-03	2.27E-02	C26H45NO6S	HMDB0000951	Bile acids, alcohols and derivatives	Lipids and lipid-like molecules	1
Taurallocholic acid	514.2852	10.29	T3-ESI(−)	3.1	3.23	1.27E-03	1.49E-02	C26H45NO7S	HMDB0000922	Bile acids, alcohols and derivatives	Lipids and lipid-like molecules	1
Glycoursodeoxycholic acid	448.3068	11.12	T3-ESI(−)	2.5	2.41	2.29E-02	9.32E-02	C26H43NO5	HMDB0000708	Bile acids, alcohols and derivatives	Lipids and lipid-like molecules	1
Chenodeoxycholic acid glycine conjugate	448.3080	12.75	T3-ESI(−)	1.8	2.67	1.26E-02	6.48E-02	C26H43NO5	HMDB0000637	Bile acids, alcohols and derivatives	Lipids and lipid-like molecules	1
Glycocholic acid	464.3023	11.11	T3-ESI(−)	1.7	2.07	3.15E-02	1.17E-01	C26H43NO6	HMDB0000138	Bile acids, alcohols and derivatives	Lipids and lipid-like molecules	1
Gamma-glutamylvaline	245.1143	2.81	T3-ESI(−)	1.0	1.38	4.12E-02	1.40E-01	C10H18N2O5	HMDB0011172	Amino acids, peptides, and analogues	Organic acids and derivatives	1
Aspartylphenylalanine	281.1145	4.14	T3-ESI(+)	2.1	3.54	1.28E-02	6.52E-02	C13H16N2O5	HMDB0000706	Amino acids, peptides, and analogues	Organic acids and derivatives	1
L-arginine	175.1199	0.66	T3-ESI(+)	2.1	1.80	8.99E-05	2.53E-03	C6H14N4O2	HMDB0000517	Amino acids, peptides, and analogues	Organic acids and derivatives	1
Phenylalanine	164.0723	2.93	T3-ESI(−)	1.1	1.87	6.01E-06	4.17E-04	C9H11NO2	HMDB0000159	Amino acids, peptides, and analogues	Organic acids and derivatives	1
Sphingosine	300.2900	13.99	T3-ESI(+)	1.9	0.55	7.07E-03	4.59E-02	C18H37NO2	HMDB0000252	Amines	Organic nitrogen compounds	1
Lenticin	247.1441	4.63	T3-ESI(+)	2.0	1.62	2.64E-02	1.02E-01	C14H18N2O2	HMDB0061115	Amino acids, peptides, and analogues	Organic acids and derivatives	1
Phenyllactic acid	165.0565	6.31	T3-ESI(−)	4.1	12.43	2.02E-10	2.91E-07	C9H10O3	HMDB0000779		Benzenoids	1

Table S4 Annotated significantly different metabolites in the serum of SFTS patients (continued).

Name	*m/z*	RT	Mode	VIP	FC	*P*-value	FDR	Formula	HMDB ID	Subclass	Superclass	Level
Hydroxyphenyllactic acid	181.0510	4.14	T3-ESI(−)	2.4	3.46	3.98E-08	1.05E-05	C9H10O4	HMDB0000755		Phenylpropanoids and polyketides	1
Gluconic acid	195.0520	0.69	T3-ESI(−)	3.9	26.15	8.11E-08	1.60E-05	C6H12O7	HMDB0000625	Carbohydrates and carbohydrate conjugates	Organic acids and derivatives	1
Proline betaine	144.1022	0.88	T3-ESI(+)	3.4	0.28	7.00E-04	9.67E-03	C7H13NO2	HMDB0004827	Amino acids, peptides, and analogues	Organic acids and derivatives	1
Cytosine	112.0507	0.91	T3-ESI(+)	3.8	0.22	7.68E-07	7.82E-05	C4H5N3O	HMDB0000630	Pyrimidines and pyrimidine derivatives	Organoheterocyclic compounds	1
O-palmitoleoylcarnitine	398.3263	14.73	T3-ESI(+)	2.7	2.34	8.29E-04	1.12E-02	C23H43NO4	HMDB0240782	Fatty acid esters	Lipids and lipid-like molecules	2
O-(17-carboxyheptadecanoyl)carnitine	458.3503	13.03	T3-ESI(+)	1.1	0.71	2.61E-02	1.02E-01	C25H47NO6	HMDB0240777	Fatty acid esters	Lipids and lipid-like molecules	2
LysoPC(0:0/16:0)	496.3432	15.77	T3-ESI(+)	1.4	1.81	2.27E-04	4.43E-03	C24H50NO7P	HMDB0240262	Glycerophosphocholines	Lipids and lipid-like molecules	2
Epiandrosterone sulfate	369.1743	12.21	T3-ESI(−)	1.1	1.51	4.09E-02	1.39E-01	C19H30O5S	HMDB0062657	Sulfated steroids	Lipids and lipid-like molecules	2
LysoPI(16:0/0:0)	571.2891	17.21	T3-ESI(−)	1.1	0.53	3.48E-02	1.25E-01	C25H49O12P	HMDB0061695	Glycerophosphoinositols	Lipids and lipid-like molecules	2
Glucose-1,3-mannose oligosaccharide	365.1078	0.73	T3-ESI(+)	2.5	0.45	1.62E-02	7.58E-02	C12H22O11	HMDB0060068	Carbohydrates and carbohydrate conjugates	Organic oxygen compounds	2
beta-D-Arabinopyranose	149.0459	0.75	T3-ESI(−)	3.1	5.32	1.30E-07	2.36E-05	C5H10O5	HMDB0029942	Carbohydrates and carbohydrate conjugates	Organic oxygen compounds	2

Table S4 Annotated significantly different metabolites in the serum of SFTS patients (continued).

Name	*m/z*	RT	Mode	VIP	FC	*P*-value	FDR	Formula	HMDB ID	Subclass	Superclass	Level
LysoPC(17:0/0:0)	510.3560	16.89	T3-ESI(+)	3.2	2.81	9.33E-05	2.59E-03	C25H52NO7P	HMDB0012108	Glycerophosphocholines	Lipids and lipid-like molecules	2
N2-gamma-glutamylglutamine	274.1046	0.72	T3-ESI(−)	1.1	0.58	4.56E-02	1.49E-01	C10H17N3O6	HMDB0011738	Amino acids, peptides, and analogues	Organic acids and derivatives	2
LysoPE(22:5(7Z,10Z,13Z,16Z,19Z)/0:0)	526.2943	15.95	T3-ESI(−)	1.0	1.61	4.24E-02	1.43E-01	C27H46NO7P	HMDB0011525	Glycerophosphoethanolamines	Lipids and lipid-like molecules	2
LysoPE(20:4(5Z,8Z,11Z,14Z)/0:0)	502.2953	15.50	T3-ESI(+)	1.1	0.60	2.57E-03	2.36E-02	C25H44NO7P	HMDB0011517	Glycerophosphoethanolamines	Lipids and lipid-like molecules	2
LysoPE(18:1(9Z)/0:0)	478.2948	16.56	T3-ESI(−)	1.0	1.79	1.48E-02	7.23E-02	C23H46NO7P	HMDB0011506	Glycerophosphoethanolamines	Lipids and lipid-like molecules	2
LysoPE(18:1(11Z)/0:0)	478.2935	16.24	T3-ESI(−)	1.1	1.92	9.47E-03	5.46E-02	C23H46NO7P	HMDB0011505	Glycerophosphoethanolamines	Lipids and lipid-like molecules	2
Leucylproline	229.1562	1.16	T3-ESI(+)	1.6	0.50	6.98E-05	2.23E-03	C11H20N2O3	HMDB0011175	Amino acids, peptides, and analogues	Organic acids and derivatives	2
Isoleucylproline	229.1554	0.92	T3-ESI(+)	1.6	0.49	1.38E-04	3.22E-03	C11H20N2O3	HMDB0011174	Amino acids, peptides, and analogues	Organic acids and derivatives	2
LysoPC(O-18:0/0:0)	510.3923	18.66	T3-ESI(+)	1.5	2.04	8.13E-03	4.99E-02	C26H56NO6P	HMDB0011149	Glycerophosphocholines	Lipids and lipid-like molecules	2
LysoPE(18:0/0:0)	480.3094	15.78	T3-ESI(−)	1.1	0.57	4.40E-04	6.89E-03	C23H48NO7P	HMDB0011130	Glycerophosphoethanolamines	Lipids and lipid-like molecules	2
LysoPE(0:0/18:0)	482.3265	14.86	T3-ESI(+)	1.6	2.32	1.59E-02	7.48E-02	C23H48NO7P	HMDB0011129	Glycerophosphocholines	Lipids and lipid-like molecules	2

Table S4 Annotated significantly different metabolites in the serum of SFTS patients (continued).

Name	*m/z*	RT	Mode	VIP	FC	*P*-value	FDR	Formula	HMDB ID	Subclass	Superclass	Level
LysoPC(P-16:0/0:0)	480.3455	16.69	T3-ESI(+)	1.6	2.00	4.45E-04	6.91E-03	C24H50NO6P	HMDB0010407	Glycerophosphocholines	Lipids and lipid-like molecules	2
LysoPC(18:0/0:0)	524.3748	17.70	T3-ESI(+)	1.3	1.85	2.14E-03	2.06E-02	C26H54NO7P	HMDB0010384	Glycerophosphocholines	Lipids and lipid-like molecules	2
LysoPC(16:1/0:0)	494.3252	14.55	T3-ESI(+)	2.3	1.96	3.14E-03	2.69E-02	C24H48NO7P	HMDB0010383	Glycerophosphocholines	Lipids and lipid-like molecules	2
LysoPC(15:0/0:0)	482.3268	15.21	T3-ESI(+)	1.4	1.84	1.13E-03	1.38E-02	C23H48NO7P	HMDB0010381	Glycerophosphocholines	Lipids and lipid-like molecules	2
LysoPC(14:0/0:0)	468.3113	14.30	T3-ESI(+)	1.2	1.74	6.21E-03	4.16E-02	C22H46NO7P	HMDB0010379	Glycerophosphocholines	Lipids and lipid-like molecules	2
Linoleyl carnitine	424.3425	15.18	T3-ESI(+)	1.4	1.78	3.71E-03	3.07E-02	C25H46NO4	HMDB0006469	Fatty acid esters	Lipids and lipid-like molecules	2
Phenylacetylglutamine	263.1045	5.12	T3-ESI(−)	3.0	4.06	2.08E-04	4.26E-03	C13H16N2O4	HMDB0006344	Amino acids, peptides, and analogues	Organic acids and derivatives	2
Tetradecanoylcarnitine	372.3108	14.22	T3-ESI(+)	1.5	2.13	1.03E-02	5.71E-02	C21H42NO4	HMDB0005066	Fatty acid esters	Lipids and lipid-like molecules	2
Oleoylcarnitine	426.3582	16.05	T3-ESI(+)	1.1	1.65	7.38E-03	4.71E-02	C25H48NO4	HMDB0005065	Fatty acid esters	Lipids and lipid-like molecules	2
Decadienedioic acid	197.0821	7.05	T3-ESI(−)	2.2	4.10	1.07E-02	5.77E-02	C10H14O4	HMDB0242172	Fatty acids and conjugates	Lipids and lipid-like molecules	2
1-O-Hexadecyl-lyso-sn-glycero-3-phosphocholine	482.3603	16.62	T3-ESI(+)	1.5	2.00	6.36E-04	9.05E-03	C24H52NO6P	HMDB0243890	Glycerophosphocholines	Lipids and lipid-like molecules	2

Table S4 Annotated significantly different metabolites in the serum of SFTS patients (continued).

Name	*m/z*	RT	Mode	VIP	FC	*P*-value	FDR	Formula	HMDB ID	Subclass	Superclass	Level
cis-5-Tetradecenoylcarnitine	370.2958	13.40	T3-ESI(+)	2.3	2.02	1.75E-03	1.79E-02	C21H39NO4	HMDB0002014	Fatty acid esters	Lipids and lipid-like molecules	2
Stearoylcarnitine	428.3756	17.04	T3-ESI(+)	1.9	2.43	2.80E-05	1.07E-03	C25H50NO4	HMDB0000848	Fatty acid esters	Lipids and lipid-like molecules	2
Octanoylcarnitine	288.2171	8.93	T3-ESI(+)	1.0	0.63	2.20E-02	9.09E-02	C15H29NO4	HMDB0000791	Fatty acid esters	Lipids and lipid-like molecules	2
Pregnenolone sulfate	395.1895	12.28	T3-ESI(−)	1.7	2.66	1.59E-03	1.70E-02	C21H32O5S	HMDB0000774	Sulfated steroids	Lipids and lipid-like molecules	2
O-[(9Z)-17-carboxyheptadec-9-enoyl]carnitine	456.3321	12.33	T3-ESI(+)	1.9	0.52	1.38E-03	1.55E-02	C25H45NO6	HMDB0240778	Fatty acid esters	Lipids and lipid-like molecules	2
gamma-Gutamylphenylalanine	295.1297	4.64	T3-ESI(+)	2.6	0.32	3.18E-05	1.16E-03	C14H18N2O5	HMDB0000594	Amino acids, peptides, and analogues	Organic acids and derivatives	2
2,4-Dihydroxybutanoic acid	119.0352	0.85	T3-ESI(−)	1.3	8.87	2.55E-02	1.00E-01	C4H8O4	HMDB0000360	Short-chain hydroxy acids and derivatives	Organic acids and derivatives	2
2-Hydroxy-3-methylpentanoic acid	131.0716	5.42	T3-ESI(−)	1.2	1.79	2.72E-03	2.42E-02	C6H12O3	HMDB0000317	Fatty acids and conjugates	Lipids and lipid-like molecules	2
(3-Heptadecanoyloxy-2-hydroperoxypropyl) 2-(trimethylazaniumyl)ethyl phosphate	524.3362	16.70	T3-ESI(−)	1.1	0.55	7.56E-03	4.78E-02	C25H52NO8P	-	Glycerophosphocholines	Lipids and lipid-like molecules	2
(E)-2-(2-cyclohexyl-2-oxoethyl)-3-ethylbut-2-enedioic acid	267.1247	13.01	T3-ESI(−)	1.1	1.59	2.22E-02	9.13E-02	C14H20O5	-	Medium-chain keto acids and derivatives	Organic acids and derivatives	2

Table S4 Annotated significantly different metabolites in the serum of SFTS patients (continued).

Name	*m/z*	RT	Mode	VIP	FC	*P*-value	FDR	Formula	HMDB ID	Subclass	Superclass	Level
Aminopentanoyloxy-octadecanoyloxypropyl trimethylammonium phosphate	623.4416	15.53	T3-ESI(+)	1.1	1.34	4.24E-02	1.43E-01	C31H63N2O8P	-	Glycerophosphocholines	Lipids and lipid-like molecules	2
[3-carboxy-2-[(7E,9E)-tetradeca-7,9-dienoyl]oxypropyl]-trimethylazanium	368.2797	12.58	T3-ESI(+)	1.8	2.63	4.46E-03	3.45E-02	$C21H38NO4^{+}$	-	Fatty acid esters	Lipids and lipid-like molecules	2
12,15-Dioxohexadecanoic acid	283.1918	14.14	T3-ESI(−)	1.8	2.88	6.38E-03	4.18E-02	C16H28O4	-	Fatty acids and conjugates	Lipids and lipid-like molecules	2
Acetyl dimethyl hydroxy estrane sulfate	397.2059	11.09	T3-ESI(−)	1.3	2.33	1.22E-02	6.35E-02	C21H34O5S	-	Sulfated steroids	Lipids and lipid-like molecules	2
2-(3-Phenylpropyl)butanedioic acid	235.0981	8.69	T3-ESI(−)	1.4	2.02	4.98E-02	1.58E-01	C13H16O4	-	Fatty acids and conjugates	Lipids and lipid-like molecules	2
2-Hydroxy-3-[3,4,5-trihydroxy-6-(hydroxymethyl)oxane]propyl tetradecanoate	463.2915	17.90	T3-ESI(−)	3.0	0.25	1.28E-04	3.08E-03	C23H44O9	-	Glycosylglycerols	Lipids and lipid-like molecules	2
4,13-Dioxotetradecanoic acid	255.1605	12.35	T3-ESI(−)	2.0	3.12	4.17E-03	3.32E-02	C14H24O4	-	Fatty acids and conjugates	Lipids and lipid-like molecules	2
4-O-(2-hexanoyloxyethyl) 1-O-octyl butanedioate	373.259	19.62	T3-ESI(+)	3.0	2.07	6.29E-03	4.16E-02	C20H36O6	-	Fatty alcohol esters	Lipids and lipid-like molecules	2
6-[6-(sulfoxy steroid)-2-methylheptan-3-yl]oxy-3,4,5-trihydroxy-2-carboxylic acid	657.3314	13.57	T3-ESI(−)	1.6	1.97	9.55E-03	5.47E-02	C33H54O11S	-	Steroidal glycosides	Lipids and lipid-like molecules	2
6-[oxy steroid]-3,4,5-trihydroxy-2-carboxylic acid	449.2552	15.15	T3-ESI(−)	1.5	2.06	1.62E-02	7.58E-02	C25H38O7	-	Steroidal glycosides	Lipids and lipid-like molecules	2

Table S4 Annotated significantly different metabolites in the serum of SFTS patients (continued).

Name	*m/z*	RT	Mode	VIP	FC	*P*-value	FDR	Formula	HMDB ID	Subclass	Superclass	Level
LysoPC(P-18:1/0:0)	508.3769	17.13	T3-ESI(+)	1.3	1.84	3.99E-03	3.20E-02	C26H54NO6P	HMDB0013122	Glycerophosphocholines	Lipids and lipid-like molecules	2
N-[1-(2-methyl-1,3-oxathiolan-5-yl)-2-oxopyrimidin-4-yl]acetamide	278.0572	3.97	T3-ESI(+)	3.0	0.29	2.81E-04	5.16E-03	C10H13N3O3S	-		Nucleosides, nucleotides, and analogues	2
LysoPC(0:0/14:0)	468.3112	13.95	T3-ESI(+)	2.4	2.81	1.23E-03	1.47E-02	C22H46NO7P	HMDB0010379	Glycerophosphocholines	Lipids and lipid-like molecules	2
PC(18:3/0:0)	518.3247	14.76	T3-ESI(+)	2.3	2.78	4.53E-03	3.48E-02	C26H48N1O7P	-	Glycerophosphocholines	Lipids and lipid-like molecules	2
Undec-2-enedioic acid	213.1138	8.95	T3-ESI(−)	2.6	2.81	5.48E-04	8.25E-03	C11H18O4	HMDB0340615	Fatty acids and conjugates	Lipids and lipid-like molecules	2
Bile acids, alcohols and derivatives	414.3033	12.75	T3-ESI(+)	2.0	0.38	1.33E-02	6.64E-02	C26H43NO5	-	Bile acids, alcohols and derivatives	Lipids and lipid-like molecules	3
Glycerophosphocholines	510.3561	16.73	T3-ESI(+)	2.2	2.11	1.86E-03	1.86E-02	C25H52NO7P	-	Glycerophosphocholines	Lipids and lipid-like molecules	3
Glycerophosphocholines	510.3562	17.09	T3-ESI(+)	1.4	1.86	1.31E-03	1.53E-02	C25H52NO7P	-	Glycerophosphocholines	Lipids and lipid-like molecules	3
Glycerophosphoethanolamines	500.2789	15.50	T3-ESI(−)	1.1	1.72	3.98E-03	3.20E-02	C25H44NO7P	-	Glycerophosphoethanolamines	Lipids and lipid-like molecules	3

Table S4 Annotated significantly different metabolites in the serum of SFTS patients (continued).

Name	*m/z*	RT	Mode	VIP	FC	*P*-value	FDR	Formula	HMDB ID	Subclass	Superclass	Level
Glycerophosphocholines	494.3257	14.87	T3-ESI(+)	1.0	1.57	2.02E-02	8.57E-02	C24H48NO7P	-	Glycerophosphocholines	Lipids and lipid-like molecules	3
Glycerophosphocholines	494.3271	14.98	T3-ESI(+)	1.1	1.64	1.03E-02	5.71E-02	C24H48NO7P	-	Glycerophosphocholines	Lipids and lipid-like molecules	3
Glycosylglycerols	463.2917	18.13	T3-ESI(−)	3.1	0.27	5.03E-05	1.72E-03	C23H44O9	-	Glycosylglycerols	Lipids and lipid-like molecules	3

m/z, mass-to-charge; RT, retention time; VIP, variable important in the projection; FC, fold change , the FC values represent the fold change in metabolic levels of the B_fatal group relative to the B_survival group; The p-value represents the original significance level from the t-test; the FDR (q-value) is the p-value adjusted using the False Discovery Rate method.

Table S5 The performance of single biomarkers. Train and optimize the random forest model using data from stage B. Then, use data from time point A for prediction (Table S5.1) and validate on the test dataset (Table S5.2), sorted in descending order of AUC values.

Table S5.1

Markers	AUC	Sensitivity	Specificity
Phenyllactic acid	0.79	0.70	0.57
Isocitric acid	0.78	0.70	0.60
LysoPC (P-18:0/0:0)	0.69	0.58	0.57
Phenylalanine	0.67	0.58	0.86
Sphingosine-1-phosphate	0.67	0.42	0.67
Gluconic acid	0.63	0.25	0.76
Indole-3-lactic acid	0.57	0.55	0.36

Table S5.2

Markers	AUC	Sensitivity	Specificity
Isocitric acid	0.74	0.50	0.57
Phenyllactic acid	0.72	0.75	0.64
Indole-3-lactic acid	0.72	0.50	0.71
Phenylalanine	0.70	0.50	0.86
LysoPC (P-18:0/0:0)	0.61	0.50	0.71
Gluconic acid	0.60	0.25	1.00
Sphingosine-1-phosphate	0.54	0.50	0.57

Table S6 The performance of the biomarker combination models. The table presents the AUC, sensitivity, and specificity for marker combinations of 2 to 7 markers. Combinations were formed by sequentially selecting markers with the highest individual AUCs in the discovery and test sets.

Combination	Test	AUCs	Sensitivity/<br>Specificity	Combination	Test	AUCs	Sensitivity/<br>Specificity
**M23**	**Test1**	**0.84**	0.75<br>0.71	M23467	Test1	0.87	0.58<br>0.90
	**Test2**	**0.85**	0.75<br>0.90		Test2	0.77	1.00<br>0.71
M235	Test1	0.84	0.75<br>0.71	M23567	Test1	0.83	0.75<br>0.71
	Test2	0.82	0.67<br>0.90		Test2	0.79	0.67<br>0.90
M237	Test1	0.82	0.75<br>0.79	M123467	Test1	0.79	0.67<br>0.90
	Test2	0.85	0.75<br>0.71		Test2	0.82	0.75<br>0.71
M2356	Test1	0.83	0.75<br>0.71	M123567	Test1	0.77	0.58<br>0.90
	Test2	0.81	0.75<br>0.90		Test2	0.82	0.75<br>0.71
M2367	Test1	0.82	0.75<br>0.90	M1234567	Test1	0.78	0.75<br>0.71
	Test2	0.84	0.75<br>0.71		Test2	0.80	0.58<br>0.90

Train and optimize the random forest model using data from stage B, then use data from stage A for prediction (test1) and validate on the test dataset (test2).

M1, Gluconic acid; M2, Phenyllactic acid; M3, Isocitric acid; M4, Sphingosine-1-phosphate; M5, Indole-3-lactic acid; M6, Phenylalanine; M7, LysoPC(P-18:0/0:0)

Table S7 Associations between prognostic biomarkers and patient parameters. Pearson or Spearman correlation was used depending on data distribution (normal or non-normal, respectively).

	**Viral load**	**WBC**	**RBC**	**HGB**	**PLT**	**HCT**	**RDW**	**MPV**	**AST/ALT**	**TBIL**	**A/G**	**TBA**	**SOD**	**BUN**	**CREA**
Phenyllactic acid	0.71 **	-0.20	0.11	0.01	-0.55 **	0.00	0.21	0.17	6.53 **	-0.01	-0.05	0.51 *	-0.16	0.43 **	0.60 **
Isocitric acid	0.34 **	0.03	0.05	0.07	-0.26 *	-0.00	0.05	-0.01	0.41 **	0.04	0.11	0.25 **	-0.14	0.28 *	0.39 **

	**CYSC**	**CKMB-IM**	**hsCRP**	**GGT**	**IL6**	**ApoA**	**LDH**	**HSTNI**	**PT**	**PTTA**	**APTT**	**TT**	**FIB**	**DD**
Phenyllactic acid	0.64 **	0.32	0.60 **	-0.03	0.65 **	-0.25	0.60 **	0.35 **	0.22	-0.25	0.67 **	0.57 **	-0,36 **	0.47 **
Isocitric acid	0.40 **	0.26	0.13	0.13	0.40 **	-0.11	0.12	0.07	0.04	-0.12	0.27 *	0.22	-0.04	0.29 *

*, $0.01 < p < 0.05$; **, $0.001 < p < 0.01$

Abbreviations: WBC: white blood cell; RBC: red blood cell; HGB: hemoglobin; PLT: platelet; HCT: hematocrit; RDW: red cell distribution width; MPV: mean platelet volume; AST/ALT: aspartate transaminase / alanine transaminase; TBIL: total bilirubin; A/G: albumin/globulin ratio; TBA: total bile acid; SOD: superoxide dismutase; BUN: blood urea nitrogen; CREA: creatinine; CYSC: cystatin C; CKMB-IM: creatine kinase-MB Isoenzyme; hsCRP: high-sensitivity C-reactive protein; GGT: gamma-glutamyl transferase:; IL6: interleukin; ApoA: apolipoprotein A; LDH: lactate dehydrogenase; HSTNI: high-sensitivity troponin I; PT: prothrombin time; PTTA: partial thromboplastin time activated; APTT: activated partial thromboplastin time; TT: thrombin Time; FIB: fibrinogen; DD: D-dimer.

Figure S1 Duration of illness for each stage (A, B, C). Based on clinical presentations, the data were then classified into 3 groups according to SFTS stage from the onset of symptoms to outcomes: stage A (0–8 days), stage B (6–15 days), stage C (12–21 days).

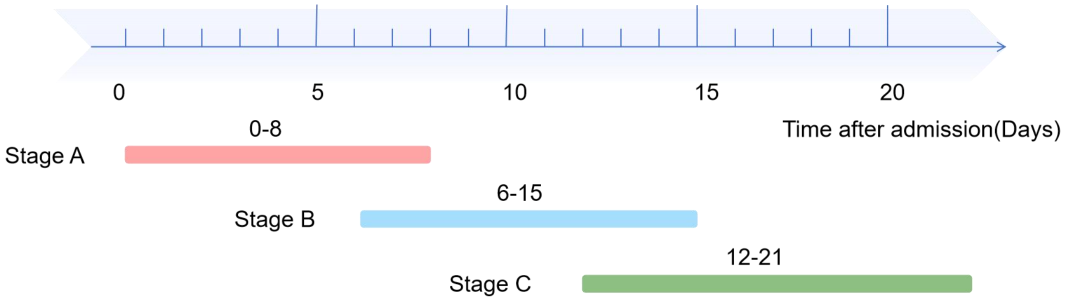

Figure S2 Cross-validation plot with a permutation test repeated 200 times of the PLS-DA score plot. (A) The PLS-DA model at three distinct time points during disease progression in SFTS patients who recovered. (B) The PLS-DA model between the fatal and survival groups of SFTS patients.

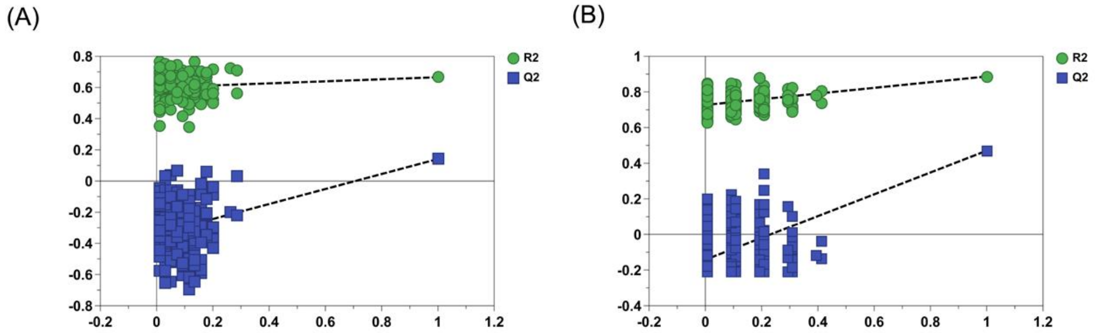

Figure S3 A hierarchical clustering heatmap of 88 annotated differential serum metabolites comparing the fatal and survival groups in SFTS patients.

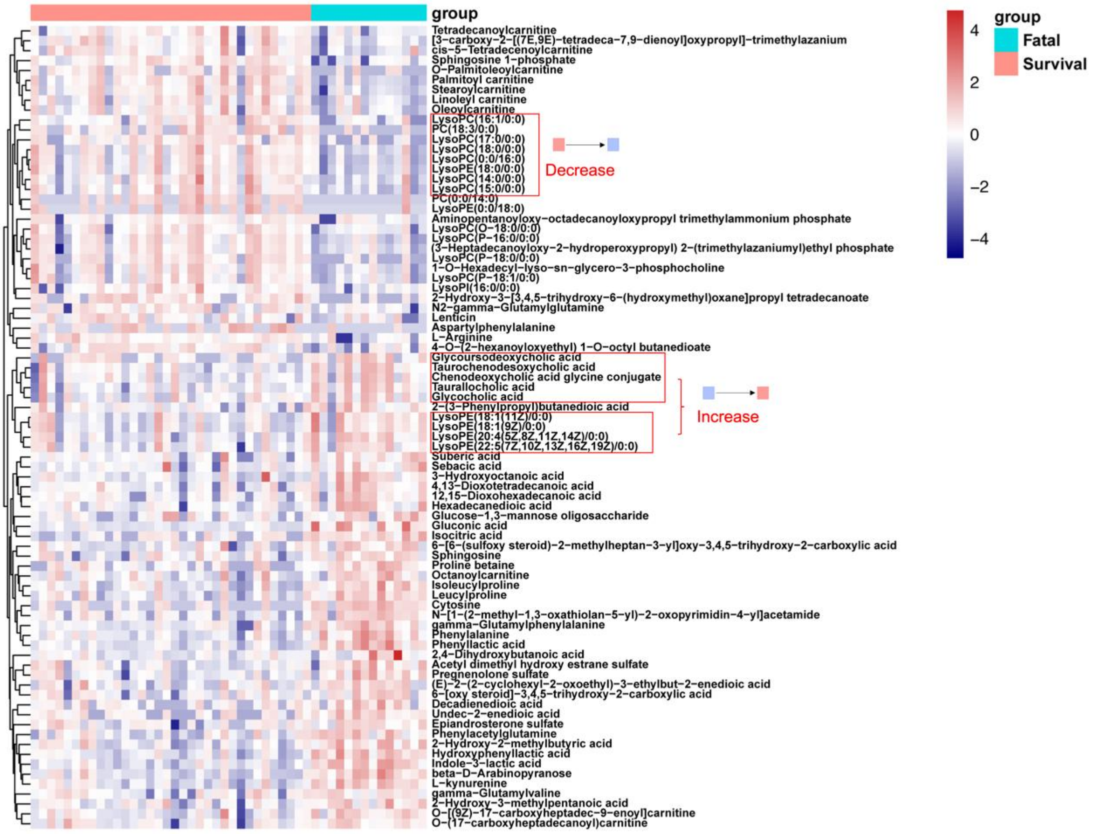

Figure S4 Comparison of potential biomarker profiles across three stages of disease progression in SFTS patients. At each stage, patients were categorized into survival and fatal groups to observe trends. The concentration trends of metabolites between these two groups remained consistent throughout all three time points.

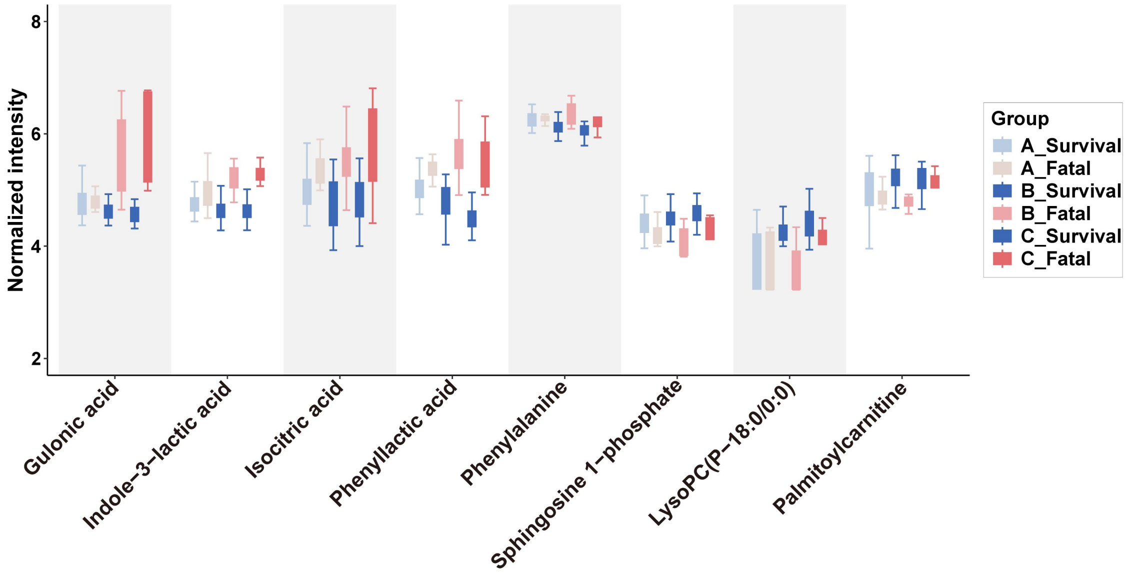

Figure S5 The trends of potential metabolic biomarkers in the test set of patients. (A) Intensity profiles of potential metabolic biomarkers between the survival and fatal groups. (B) Intensity profiles of potential metabolic biomarkers across three stages of disease progression. In line with the discovery set, metabolites with higher concentrations in the fatal group showed a gradual decrease in the survival group over the three stages. Conversely, metabolites with lower concentrations in the fatal group exhibited a gradual increase over time in the survival group.

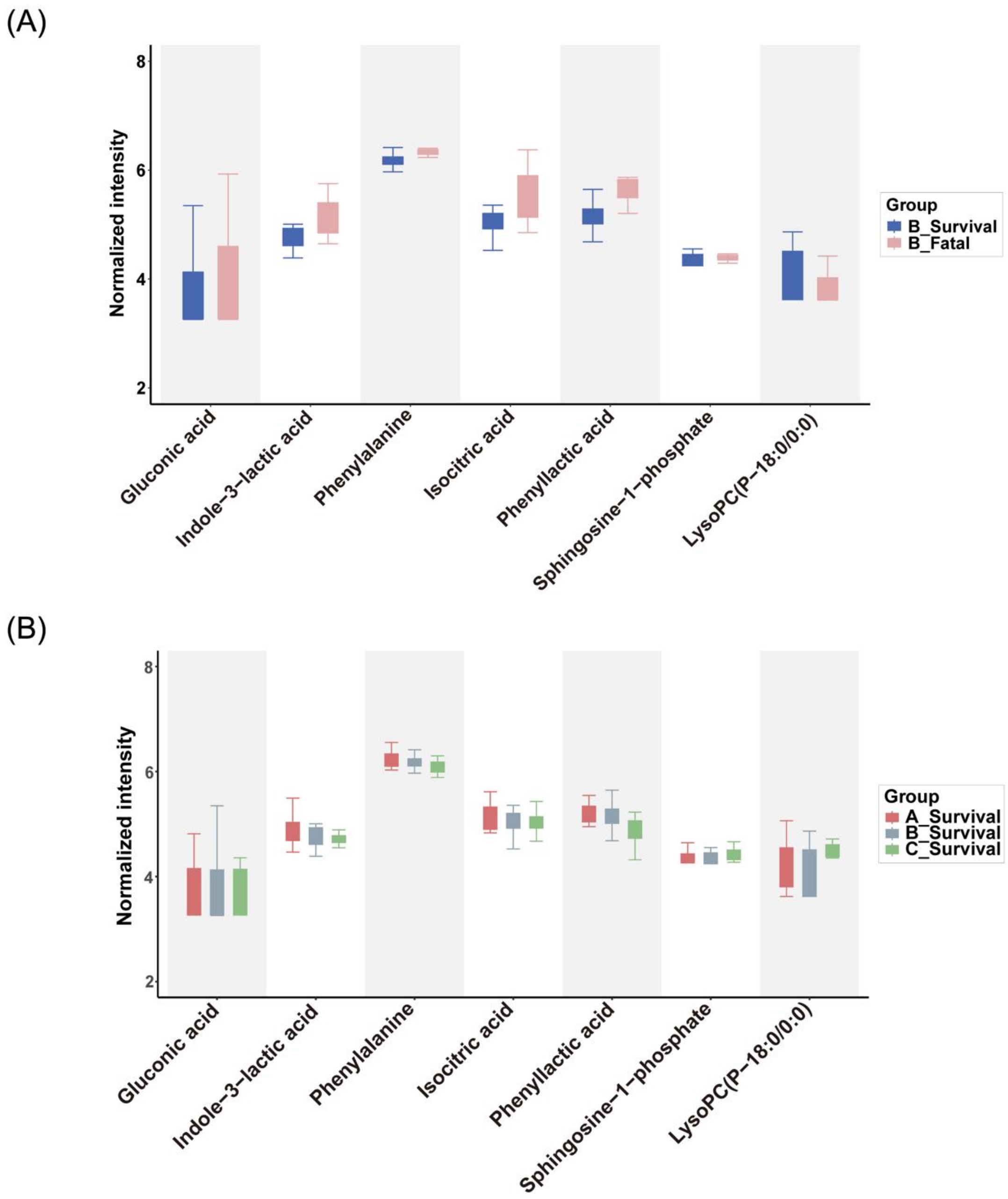

Figure S6 ROC curves for Age (AUC = 0.73), BUN (AUC = 0.65), and APTT (AUC = 0.78).

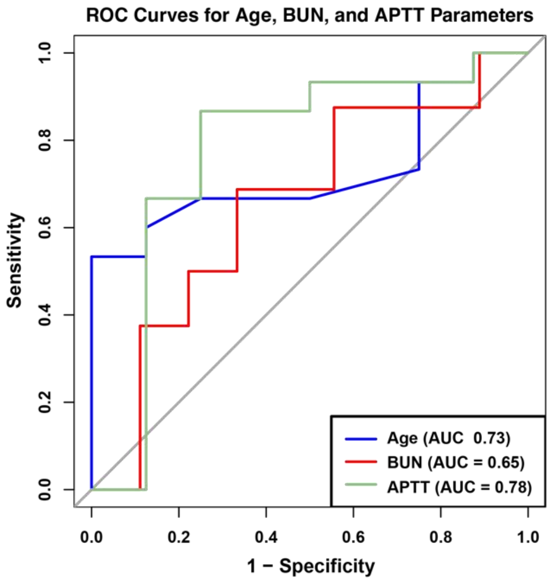

Figure S7 The patient scores derived from the metabolic prognostic model output. Higher output scores indicate a worse prognosis.

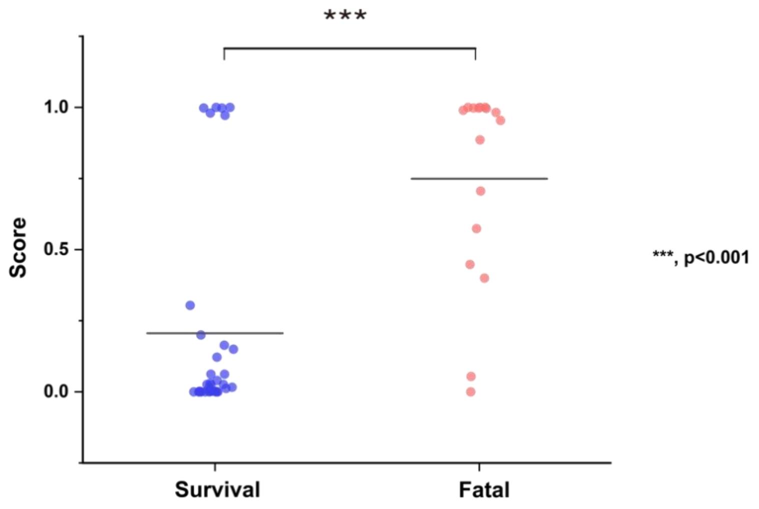

Supplement: Supplementary file 1 [file metabolites-15-00228-s001.zip › metabolites-3476317-supplementary.pdf]
